# AAPM Medical Physics Practice Guideline 8.b: Linear accelerator performance tests

**DOI:** 10.1002/acm2.14160

**Published:** 2023-10-04

**Authors:** Robert F. Krauss, Salim Balik, Eileen T. Cirino, Austin Hadley, Navneeth Hariharan, Shannon M. Holmes, Kayla Kielar, Hossein Lavvafi, Kiernan McCullough, Steven Palefsky, J. Phillip Sawyer, Koren Smith, Jason Tracy, Jeff D. Winter, Nicholai E. Wingreen

**Affiliations:** ^1^ St. Francis Hospital Memphis Tennessee USA; ^2^ University of Southern California Los Angeles California USA; ^3^ Beth Israel Lahey Health Burlington Massachusetts USA; ^4^ Anchorage Radiation Oncology Center Anchorage Alaska USA; ^5^ Standard Imaging, Inc. Middleton Wisconsin USA; ^6^ Varian Medical Systems Palo Alto California USA; ^7^ CTSI Oncology Solutions Washington District of Columbia USA; ^8^ Colorado Associates in Medical Physics Colorado Springs Colorado USA; ^9^ Elekta Atlanta Georgia USA; ^10^ Sawyer Medical Physics LLC Cincinnati Ohio USA; ^11^ UMass Chan Medical School/IROC Rhode Island QA Center Lincoln Rhode Island USA; ^12^ Sun Nuclear Melbourne Florida USA; ^13^ Department of Medical Physics Princess Margaret Cancer Centre Toronto Ontario Canada; ^14^ American Association of Physicists in Medicine Alexandria Virginia USA

**Keywords:** baseline, commissioning, external beam, guideline, IMRT, linac, MPPG, percentage depth dose, primary reference data, profiles, quality assurance, TG‐142, TG‐198, VMAT

## Abstract

The purpose of this guideline is to provide a list of critical performance tests to assist the Qualified Medical Physicist (QMP) in establishing and maintaining a safe and effective quality assurance (QA) program. The performance tests on a linear accelerator (linac) should be selected to fit the clinical patterns of use of the accelerator and care should be given to perform tests which are relevant to detecting errors related to the specific use of the accelerator. Current recommendations for linac QA were reviewed to determine any changes required to those tests highlighted by the original report as well as considering new components of the treatment process that have become common since its publication. Recommendations are made on the acquisition of reference data, routine establishment of machine isocenter, basing performance tests on clinical use of the linac, working with vendors to establish QA tests and performing tests after maintenance and upgrades. The recommended tests proposed in this guideline were chosen based on consensus of the guideline's committee after assessing necessary changes from the previous report. The tests are grouped together by class of test (e.g., dosimetry, mechanical, etc.) and clinical parameter tested. Implementation notes are included for each test so that the QMP can understand the overall goal of each test. This guideline will assist the QMP in developing a comprehensive QA program for linacs in the external beam radiation therapy setting. The committee sought to prioritize tests by their implication on quality and patient safety. The QMP is ultimately responsible for implementing appropriate tests. In the spirit of the report from American Association of Physicists in Medicine Task Group 100, individual institutions are encouraged to analyze the risks involved in their own clinical practice and determine which performance tests are relevant in their own radiotherapy clinics.

1

The American Association of Physicists in Medicine (AAPM) is a nonprofit professional society whose primary purposes are to advance the science, education, and professional practice of medical physics. The AAPM has more than 8000 members and is the principal organization of medical physicists in the United States. The AAPM will periodically define new practice guidelines for medical physics practice to help advance the science of medical physics and to improve the quality of service to patients throughout the United States. Existing medical physics practice guidelines will be reviewed for the purpose of revision or renewal, as appropriate, on their fifth anniversary or sooner. Each medical physics practice guideline represents a policy statement by the AAPM, has undergone a thorough consensus process in which it has been subjected to extensive review, and requires the approval of the Professional Council. The medical physics practice guidelines recognize that the safe and effective use of diagnostic and therapeutic radiology requires specific training, skills, and techniques, as described in each document. Reproduction or modification of the published practice guidelines and technical standards by those entities not providing these services is not authorized. The following terms are used in the AAPM practice guidelines:

- Must and Must Not: Used to indicate that adherence to the recommendation is considered necessary to conform to this practice guideline. **While must is the term to be used in the guidelines, if an entity that adopts the guideline has shall as the preferred term, the AAPM considers that must and shall have the same meaning**.
- Should and Should Not: Used to indicate a prudent practice to which exceptions may occasionally be made in appropriate circumstances.

## 2 INTRODUCTION

Performance testing of a linear accelerator (linac) is a critical component of a comprehensive quality management program in a radiotherapy clinic that utilizes external beam photon or electron radiation therapy. The qualified medical physicist (QMP) must establish routine testing to ensure that the linac's current performance has not deviated from the clinical parameters acquired at the time of acceptance and commissioning of the machine. The QMP must further ensure that the beam models in the treatment planning system (TPS) are still appropriate for the current use of the linac.

The technology and control systems of linacs are rapidly evolving; new features emerge frequently to improve the accuracy and efficiency of treatments. The specific choice and use of technology on a linac will depend on the types of diseases treated, the clinical workload, and the workflow that has been implemented. Performance tests on each linac must be selected to fit the clinical patterns of use of the accelerator, and care must be given to selecting and performing tests that are relevant to detecting errors related to the specific use of the accelerator.

## 3 GOALS AND RATIONALE

This document is a 5‐year update to part of a series of medical physics practice guidelines commissioned by the American Association of Physicists in Medicine (AAPM) intended to describe acceptable standards for various aspects of clinical medical physics. The implementation of comprehensive quality assurance (QA) programs recommended in AAPM Task Group Reports^1^, ^2^, ^3^, ^4^ is a necessary part of operating a radiation therapy program, and the purpose of this guideline is to provide a list of critical performance tests that assist the QMP in establishing and maintaining a safe and effective QA program appropriate for the clinical use of the accelerator. This list is updated here from the previous version^5^ to reflect the changing technologies and practices in the modern radiotherapy environment. The QMP is ultimately responsible for choosing and implementing appropriate tests and plays a key role in establishing a culture of safety.

This guideline acknowledges the work of MPPG 8.a while also updating the rationale used in the review of current protocols and task groups. A thorough discussion of this guideline with respect to the original document is found in Section 5.1.

This report describes dosimetry, mechanical, and safety tests for C‐arm type linacs only. The scope of this guideline does not include tests for on‐board imaging equipment. Advancements in software and automation have made electronic portal imaging detector (EPID) systems an integral part of many linac QA programs, and thus they must be rigorously tested to ensure any QA data acquired by the imaging system is accurate. These tests are addressed in previous reports.^3^, ^4^, ^6^, ^7^

General implementation guidance for each recommended test is included so that the QMP can understand the overall goal of each test. However, this guideline is not intended to be a tutorial. In some cases, suggestions on what types of devices are helpful and suitable for measurement are made, but the choice of measurement equipment and technique is ultimately the responsibility of the QMP.

## 4 INTENDED USERS

The intended users of this report are QMPs who are conducting linac performance tests or those who are designing a QA program for linacs and seek to understand the critical tests needed to detect errors and ensure safe and high‐quality external beam radiation therapy delivery.

Administrators, linac manufacturers, personnel representing accrediting bodies, and state regulators are also encouraged to use this guideline as a reference in understanding an institution's use of equipment and necessary tests chosen by the QMP to maintain the equipment at a minimum level required to provide adequate care.

## 5 STAFF QUALIFICATIONS AND RESPONSIBILITIES

A Qualified Medical Physicist is described by AAPM Position Statement 7, MPPG 10, the ACR‐AAPM Technical Standard For the Performance of Radiation Oncology Physics for External‐Beam Therapy and other documents.^8^, ^9^, ^10^ The QMP shall be able to independently perform all of the required duties in the field of therapeutic medical physics, including designing and maintaining an overall QA Program.

The QMP must design and direct all QA activities, aid in the performance of tests or analysis if needed, and assume professional responsibility for the work done.^11^ The QMP may delegate certain QA responsibilities to qualified personnel. The QMP is responsible for reviewing and communicating the outcome of tests and ensuring that the results meet set action limits.

## 6 PERFORMANCE TEST REVIEW

### 6.1 Risk assessment: MPPG 8.a methodology

A review of both the failure mode and effects analysis (FMEA) done by the previous authors and recent publications describing risk analysis strategies for linac QA tests provided context for the revisions to MPPG 8.a.^5^ The MPPG 8.a methodology is briefly described to clarify some questions that arose following the publication of that report. Please refer to the original document for additional details. Recommended tests in MPPG 8.a were chosen based on results from the risk analysis along with the consensus of the committee at that time. Scoring participants assigned occurrence (O), lack of detectability (D), and severity (S) scores to performance tests using their experience of failure rates for the clinical parameter in question.

In some cases, the committee chose to include lower ranking tests because of safety or regulatory reasons. For example, the beam‐on indicator (daily test) scored one of the lowest risk priority number (RPN) values for daily tests; however, many state regulations require a beam‐on indicator. In some cases, the committee chose to exclude a test that scored highly. For example, the annual multi‐leaf collimator (MLC) leaf position repeatability was excluded because the committee considered that the monthly leaf position accuracy test was already testing this parameter on a more frequent basis.

### 6.2 MPPG 8.b methodology

The objective of this update was to discuss each test from TG‐142^3^, MPPG 8.a^5^ and TG‐198^4^ independently, consider components of the current treatment process that have become common practice since the prior report, and review current literature on linear accelerator QA. The MPPG 8.b committee chose not to repeat FMEA to emphasize the assertion of TG‐100^12^ that risk‐analysis is unique to each practice environment and requires an in‐depth review of the institution's processes. Each institution should conduct a local analysis of their linear accelerator QA program.

While risk analysis is unique to each institution, the experience of each institution is still instructive. Therefore, literature reporting risk analysis for linear accelerator QA is beneficial in assessing which tests different clinics have found important while considering their perspectives in relation to this guideline. A few examples are included here to demonstrate various strategies for assessing risk.

Faught et al.^13^ created a survey tool distributed to 2000 medical physicists worldwide who participate in the quality services of IROC Houston. A panel identified 11 different intensity modulated radiation therapy (IMRT) delivery mode failures at or just outside of the action limits based on TG‐142 criteria. The respondents were asked to assign the three FMEA scores (O, D, S) for each failure. Based on 184 responses, the risk was not agreed upon by the medical physics community. The authors concluded that MPPGs should provide guidance when optimizing a QA program based on the relatively new TG‐100 strategies.

O'Daniel et al.^14^ used an FMEA methodology focused on daily QA tests and their clinical impact. Each evaluation of a TG‐142 recommended daily QA test assumed an undetected test failure. Severity was scored based on modeling the error in the TPS. Occurrence was scored based on QA records. The error was assumed to be impossible to detect if the test was not performed. The RPN also considered the number of patients impacted based on the frequency of testing. The author suggested that the results indicated an opportunity to alter daily QA procedures at their institution.

Bonfantini et al.^15^ applied FMEA to all daily, monthly, and annual QA tests for three linacs individually. The analysis considered the clinical use of each linac. Each test was considered a potential failure mode. Scoring was based on two approaches: 1) a survey of five medical physicists with different levels of experience and 2) a semi‐quantitative analysis including the simulation of failures using the TPS, literature review and QA data analysis. The authors concluded that FMEA analysis is a useful tool for optimization of linac QA, but the approach should be supported with a quantitative analysis for a more robust risk evaluation.

Hosiak et al.^16^ described a method of using vendor‐provided cloud‐based machine log system to identify trends, reliability, and utilization per linac that could be used to optimize QA tests and frequency.

We recommend using the tests and frequencies outlined in this report as a minimum for any risk analysis performed and that full consideration be given to the implication of removing any of the recommended minimum components.

### 6.3 Working toward risk assessment; WG‐100 collaboration

Each center should conduct their own risk analysis of their site‐specific QA procedures. Published RPN numbers should not be used as absolute values quantifying risk as explained in TG‐100^12^ but rather as a guide for the risk analysis process, since each center has unique features within their processes. Towards that aim, the MPPG 8.b committee collaborated with the Work Group on the Implementation of TG‐100 (WG‐100) to provide guidance on creating a robust QA program based on FMEA beyond the guidance addressed in this document. Appendix I includes links to the WG‐100 website that includes both tools and a data repository. Table 1 describes a scenario for facilities with similar equipment but varying practice patterns as a simple example. The QMP is presented with two scenarios in which one facility treats IMRT prostate only and does not use the 6 degrees of freedom (DOF) couch and another facility that treats cranial stereotactic radiosurgery (SRS) using a 6DOF couch. This illustrates that the process and degree to which a couch is used varies between a prostate and general stereotactic treatment. The table shows variability in both the flow of each process and the systems’ interdependence with imaging systems for patient setup. This should serve as a general guide for QMPs to analyze their facilities’ processes to create a risk‐informed quality management program based on TG‐100 and to show that risk assessment is feasible for each clinic.

**TABLE 1:** Comparison of prostate and SRS process steps between a moderately hypofractionated prostate case and cranial SRS.

Process steps	Prostate only clinic setup	SRS setup
1	Check‐in patient and perform time out	Check‐in patient and perform time out
2	Setup patient in TX position using vac‐loc	Setup patient using SRS mask
3	Move patient to tattoos to get to ISO	Move patient to marks on mask to get to ISO
4	Perform cone‐beam computed tomography (CBCT) and 3D match	Perform CBCT and 3D match
5	Adjust patient position based on imaging and shifting only along the longitudinal, lateral, and vertical axis (3DOF) out of the available 6DOF.	Adjust patient position using 6DOF couch
6	Complete treatment	Additional imaging maybe required for mid treatment or couch kicks
7		Complete treatment

For example, in the table above, step 6 determines the type of testing required for the couch. Testing the couch longitudinal, lateral, and vertical digital readout versus measuring tape/ruler may be sufficient for the first case but the second case will require a phantom that can test the accuracy of all six degrees of couch freedom with greater precision than a ruler.

### 6.4 Leveraging data—RO‐ILS collaboration

Peer reviewed literature such as the examples presented in Section 5.2 demonstrate the benefit of tracking errors and equipment failures when assessing the frequency and severity of potential QA or equipment failures rather than using estimates or personal judgment. This guideline endorses these error and failure tracking processes and further recommends that institutions participate in a patient safety group to broaden the reporting of incidents/failures to benefit all and to support future institutional decisions. To this end, the Radiation Oncology Healthcare Advisory Council (RO‐HAC) searched the Radiation Oncology Incident Learning System (RO‐ILS) database and reported on any incidents that could impact recommendations regarding the need for and/or frequency of a certain test and to identify any rising risks associated with aspects of the QA process. RO‐ILS is one of several incident reporting systems that can be used. RO‐HAC, in conjunction with Clarity PSO, a federally listed patient safety organization (PSO) under the Patient Safety and Quality Improvement Act of 2005, has published a RO‐ILS Equipment Quality Assurance Themed Report. It is available here: https://www.astro.org/ASTRO/media/ASTRO/Patient%20Care%20and%20Research/PDFs/ROILS_TR_EquipmentQA.pdf.^17^ This guideline addresses the key findings, but the QMP should review the complete report.

### 6.5 Relative risk compared to other clinical processes

Failures in hardware and software systems on a linac are inevitable and to be expected during the life of the unit. Therefore, the QMP must design a QA program that includes tests designed to detect these failures. However, hardware and software system functions on a linac represent just one portion of the extensive process map that comprises the external beam treatment paradigm.^18^ The relative risks of hardware and software errors are lower than risks due to human process‐related errors, lack of standardized procedures, and inadequate training of staff.^12^, ^19^ While QMPs must be diligent to ensure that risks of hardware and software errors are kept low and have a minimal effect on the overall goal of delivering dose to the target with a high degree of accuracy,^20^, ^21^ the linac performance testing portion of QA programs must be efficient so that time and resources can be dedicated to other areas where risk analysis indicates errors with a higher score can also occur.^22^

## 7 MINIMUM REQUIRED RESOURCES AND EQUIPMENT

While this guideline does not endorse specific tools or techniques for performing each test, it provides guidance on methods to achieve the goal of the test. The test procedure and equipment utilized must be capable of accurately measuring to the precision required by the action limit. Tests in this guideline can be performed using the most basic tools available to the QMP. However, a wide variety of more sophisticated equipment and software tools exist to aid the QMP in performing, analyzing, and interpreting measurements accurately and efficiently. They can be costly, but actually represent a small percentage of the revenue generated by a single linear accelerator over its lifetime. The total cost of ownership for a linac must include not only the purchase price and the operating cost for the machine itself, but the appropriate quality assurance equipment, software, and associated service, as determined by a QMP.

The QMP must collaborate with administrators and managers in realizing the cost‐benefit of purchasing these tools. The quality assurance tools available must reflect the types of treatments offered at each clinic. While often improving efficiency, the most important aspects of investing in appropriate quality assurance equipment is the improved ability to meet regulatory requirements, introduction of more complex treatment techniques, and, most importantly, improved patient safety and treatment quality. In general, the QMP is uniquely qualified to assist administrators in determining what QA equipment is appropriate. However, this should be a collaborative effort between administrators and physicists, physics assistants, therapists and any other stakeholders that perform and/or assess linac QA. Finally, some quality control measures are more effective than others^23^ and the QMP must allocate the appropriate amount of time to testing each item or process relative to the risks involved.

For specific instances where either the experience of the committee has shown a consistent deficiency in the available tools provided to the QMP or tests have changed enough in this guideline to significantly increase the workload using traditionally basic equipment, as part of the guideline, the committee appeals to administrators to provide the appropriate software and/or devices. Similarly, for instances in which the committee and, by extension, QMPs feel vendors have not provided adequate tools to perform certain tests or machines that cannot consistently meet the action limits, we have included text that appeals directly to the vendors to provide the appropriate equipment and/or tests. QMPs must continue to inform vendors of any inadequacies in available tests and equipment.

The QMP and administrators must also consider the staffing requirements to perform the testing and analysis in the clinic's quality assurance program. Medical physicists, medical physicist assistants, dosimetrists, therapists, and others may all be delegated machine quality assurance duties. These must be taken into account when staffing a department. Staffing must also be reassessed any time a QA procedure changes, whether in process or equipment. ASTRO, ACR, ACRO, and other professional organizations all provide staffing recommendations.^24^, ^25^, ^26^ Some radiation oncology vendors provide staffing assessments as part of a broader workflow analysis, which often includes at least some QA processes, designed to facilitate the best use of their products.

## 8 DEVELOPING A QUALITY MANAGEMENT PROGRAM FOR PERFORMANCE TESTS

### 8.1 Primary and secondary reference data

#### 8.1.1 Primary reference data

Detection of most linac performance problems requires comparison to some primary reference dataset (PRD). Given that clinical treatment decisions are primarily based on dose calculations done in a TPS, it is best to compare ongoing linac dosimetric performance to the site's well‐commissioned clinical TPS models or TPS calculations following the principles established in MPPG 5.^27^ This ensures that the link between what the physician approves in the TPS and what the linac actually delivers is validated. Therefore, monitoring any dosimetric deviation of the linac from the same beams calculated in the TPS is of primary interest.

This approach deviates from the traditional method (seen in AAPM publications as recently as TG‐198^4^) of comparing measurement results to the linac commissioning data. The approach of using linac commissioning data as the PRD goes back as far as one of the original guides to external beam QA, AAPM report 13.^28^ However, this approach was designed in an era of hand calculations and profile transparencies that directly used the tabulated linac commissioning data to calculate beam time or monitor units (MUs).^29^ This idea was carried forward in TG‐40^2^, TG‐142,^3^ and others. But today, monitor units are calculated based on sophisticated calculation algorithms that model the linac commissioning data. Therefore, the linac commissioning data are used only indirectly in calculating dose and monitor units. In the spirit established in these earlier documents, where linac performance was tested relative to the data directly used to calculate monitor units (hand calculations based off tabulated linac commissioning data), this committee believes it is appropriate to use the TPS models (and by extension, TPS calculations) as the PRD. This must be done for every beam parameter used by the TPS to calculate the beam, such as beam profiles/off axis factors (OAFs) and percent depth dose (PDD)/tissue phantom ratio (TPR) values. This approach is similar to the way TG‐135 recommends Cyberknife users test beam profiles (see Test II.B.2, beam shapes).^30^ Following the commissioning and QA recommendations in MPPG 5 will therefore link the models to the linac commissioning data.

Further, since the overall clinical goal is to ensure that the delivered and calculated doses agree within 5%,^2^, ^20^ directly comparing to the data used for dose/MU calculations (the TPS model) limits the propagation of error that can occur when performance is linked to linac commissioning data instead of the model. MPPG 5 allows for a 2% difference between certain calculated and measured TPS parameters. Thus, if during annual testing the beam were compared to linac commissioning data, the error between annual measured data and the beam model in the TPS could be 3−4% (depending on which document's action limit was used), nearly exhausting the 5% allowance.

Non‐modeled testing parameters, such as safety, mechanicals, etc., must be compared to absolute standards.

*Note: Given that the PRD is the treatment planning system for dosimetric parameters, in order to successfully implement MPPG 8, the QMP must understand the accuracy and limitations of the TPS through a process such as implementing MPPG 5*.^27^

#### 8.1.2 Secondary reference data

**FIGURE 1:**
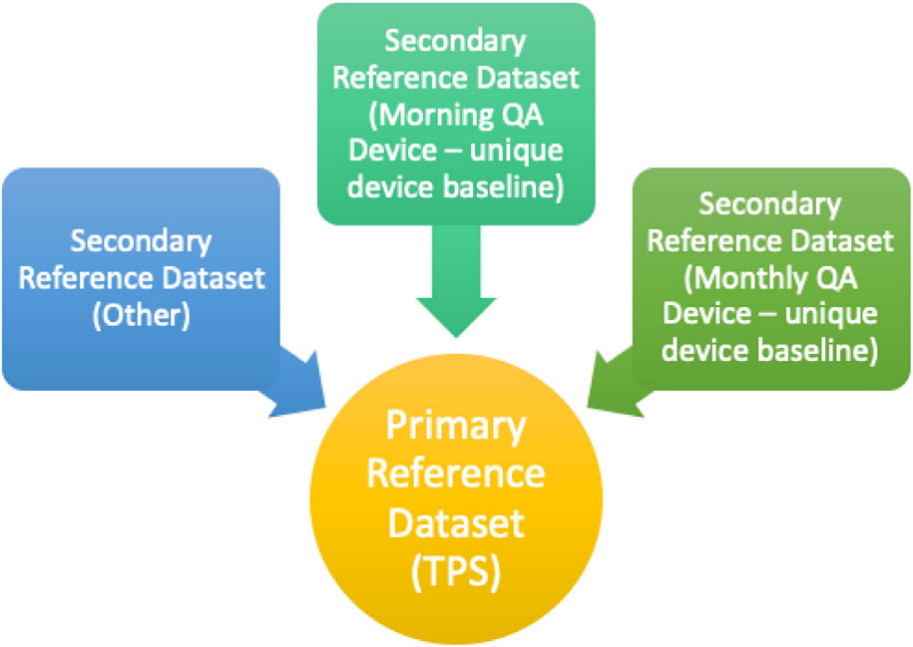
Each secondary reference dataset and/or unique device baseline that measures dosimetric parameters must refer back to the Primary Reference Dataset, the treatment planning system (TPS).

The TPS beam models discussed above are typically generated from data acquired in a water tank and, thus, a water tank may be the best choice for the most rigorous testing. An increasing number of resources have become available that also makes comparison of full array measurements to the TPS possible. It is up to the QMP to decide what method is appropriate for each beam, but the method must allow direct comparison to the TPS. However, a water tank or other appropriate system is often not an efficient choice for more routine daily or monthly measurements. For example, a secondary measurement system may be used for monthly measurements and a tertiary system may be used for daily measurements. In this case, it is necessary to create a secondary reference dataset (SRD) that has been appropriately verified against the primary reference dataset (the TPS). This secondary dataset that is linked to the PRD is thus the specific device baseline^*^ for such secondary and tertiary testing systems (Figure 1). The link between the SRD (device baseline) and the PRD must be well understood such that any deviation from the secondary reference dataset can be directly converted to a corresponding deviation from the PRD. For example, if a morning QA device measures flatness and symmetry for a 20 × 20 cm field, any daily deviation from the device baseline must allow the QMP to determine the beam deviation from the PRD. Therefore, if the annual profile measurement indicates a 1% difference from the PRD at the same measurement point and the same beam is used with the daily device, a 1% deviation from the SRD in flatness or symmetry would be equivalent to either 0 or 2% difference from the PRD (depending on the “direction” of the error, relative to the PRD). An effective approach for creating a secondary reference dataset is outlined below:

1. Perform annual beam measurements.
2. Compare results of annual measurements to TPS data or absolute standards (such as TG‐51^31^, ^32^ for absolute dose).
3. Ensure results are within the action limit (or tolerance; see Section 9) and resolve any differences.
4. Once annual beam measurements are verified, make measurements with the secondary and tertiary measurement systems to be used routinely. Ideally, this occurs in the same measurement session on the same day. The data acquired from this measurement is the SRD that is used as comparison for the secondary and tertiary measurement systems used for daily and/or monthly measurements.
5. Calculate the equivalent deviation from the PRD that is reflected in a deviation of the SRD/device baseline.

It is the responsibility of the QMP to ensure all SRDs are appropriately used and verified against the PRD (TPS) and/or absolute standards on at least an annual basis. It is also the responsibility of the QMP to ensure that all QA devices and systems are correctly and completely commissioned.

^*^The previous version of this document ^5^ *and AAPM documents such as TG‐142* ^3^ *have traditionally used the term “baseline” to refer to the primary reference dataset to which annual QA was compared. In fact, MPPG 8.a uses the terms almost interchangeably. In most cases, this dataset was the measured linac commissioning data. This was consistent with the idea that “baseline” is a set of measured data for a particular device. Since MPPG 8 has moved to using the TPS models/calculations as the PRD, it seemed misleading to continue using the term baseline since it is not actually measured. Hence, the committee has moved to calling the TPS model the PRD instead of baseline for the sake of consistency. Since secondary datasets are typically measured on the secondary/tertiary devices themselves, the idea of a “device baseline” was introduced and can usually be used interchangeably with SRD*.

### 8.2 Isocenter: establishing a reference point for the QA program

For a common C‐arm linear accelerator, the isocenter has classically been defined as the point around which the gantry, collimator, and treatment couch rotates.^33^ The coordination of these centers of rotation, or mechanical isocenters, serves as a useful reference point that allows for the precise setup of patients and equipment. Linear accelerators are equipped with numerous tools to help identify the machine isocenter (front pointers, light field crosshairs, laser systems, etc.), ensuring that treatments and testing are delivered as intended. These, however, are only approximate measures to help align the patient to the radiation isocenter of the machine, the point that the actual treatment beam rotates around. Each component that either moves the patient during treatment, changes the position of the radiation source, or collimates the radiation field will affect how accurately the machine irradiates the target.^34^ Tests such as the Winston‐Lutz or radiation star shots are methods that analyze how the center of the radiation field changes with different gantry, collimator, and couch positions with respect to a stationary target.^35^ All collimation devices must be tested separately (e.g., MLC, jaws, cone mount), as each system is aligned independently. Understanding the mechanical precision of the linear accelerator and the potential ramifications for targeting accuracy is essential when navigating clinical decision making, such as prescribing treatment margins or implementing procedures that require a high degree of accuracy.^36^

The QMP must also establish a reliable reference frame that assists physicists and therapists in aligning patients and devices to the radiation isocenter. In addition to the tools mentioned previously, systems such as surface tracking devices, on‐board imagers, and ceiling mounted x‐ray systems are only a few examples of the many options for alignment to the isocenter. The accuracy of these tools must be evaluated and action limits set according to their clinical use. Performing a Winston‐Lutz test using the preferred method of alignment allows the QMP to test the accuracy of the setup process and detect any changes in machine performance that could affect the radiation isocenter. Frequency of targeting tests must be established based on the treatment techniques employed by the clinic and their expectation of accuracy. Regular targeting tests can be instrumental in detecting changes in alignment or accuracy of setup equipment that could ultimately compromise the fidelity of patient treatment.^37^

### 8.3 Performance tests based on clinical practice

A robust QA program will be based on the needs of each clinical practice. An all‐encompassing list of linac performance tests that meets the needs of every variation in patient treatment and delivery technique does not exist. This guideline, however, provides a list of critical tests that ensures the minimum requirements to provide quality patient care.

Still, clinical practice and use of technology can vary widely on each linac and it is likely that many clinical situations will require more robust testing. The QMP is ultimately responsible for deciding which tests are prudent to perform based on the clinical need. The report from TG‐100^12^ provides excellent tools to assist the QMP with this task, while MPPG 8 serves as the minimum guideline for the body of testing suggested after following such an FMEA approach. In addition, image guidance techniques and external reference tools used to align the patient accurately for treatment on C‐arm linacs have become prevalent in clinical practice. Alignment tools that are closely tied to the mechanical aspects of a linear accelerator (i.e., imaging isocenter vs. treatment isocenter) are critically important. While not covered in this document, these tools are often used to assist in the setup of various tests in MPPG 8. Other authors suggest performance tests for alignment tools that ensure accurate positioning of the patient as well as the coincidence of the imaging and treatment isocenters.^3^, ^4^, ^5^, ^6^ The clinical application of these tools may dictate the frequency and action limits of testing. For instance, a practice in which the use of imaging to align the patient is emphasized and used more frequently than other alignment techniques, such as the lasers or the optical distance indicator (ODI), may consider decreasing the frequency of testing the lasers or ODI in favor of more robust and frequent imaging QA.

C‐arm linacs are also commonly used to treat patients with a hypofractionated SRS or stereotactic body radiation therapy (SBRT) treatment regimen.^38^, ^39^ In these instances, the QMP must refer to protocols specifically designed for performance tests in a stereotactic setting in order to achieve a higher degree of accuracy than that needed for regularly fractionated patients.^40^, ^41^, ^42^

### 8.4 Vendor provided tests and tools

Some equipment vendors provide integrated tools and test procedures that are built into the software and hardware components of a linac. These tools and tests are often valuable to the QMP, who may consider incorporating them into routine QA programs. However, it is the responsibility of the QMP to ensure that tools are independently verified to produce the desired result by an independent system before implementing into clinical use. Further, vendor tests may be a comprehensive set of tests for performance components but may not address state or institutional safety requirements. For example, they often do not include safety tests in clinical context, such as door interlock checks. Thus, the QMP must independently verify and document that vendor tests can be appropriately used to follow this guideline and supplement them when they do not span all needed characteristics of the linac QA program. In a multivendor environment, the QMP may choose to use a common test across multiple vendor machines rather than perform each of the vendors’ recommended tests. This is easier on the technical staff as they do not need to learn different tests for each machine and provides a common QA base across all machines.

The vendor should provide recommended performance and safety tests that evaluate any components unique to that particular system. Vendors best understand the design and technical challenges of their systems and are thus well equipped to create self‐testing procedures. This will assist the QMP in developing an equipment‐specific QA program most beneficial to their specific clinical practice. Just as QMPs must not rely solely on the vendor for establishment of the QA program, the vendor must not rely solely on the QMP to develop all test procedures with no vendor‐specific guidance. The vendor and the QMP must work together in the development of effective and efficient QA programs for each institution.

These vendor tests are often also based on vendor supplied tools. Some of these tools are unique to that particular machine and are required in order to complete the test. Other tools are generic, commercially available tools supplied by the vendor as part of the purchase of the machine and tied to the vendor's service procedures. It is the responsibility of the QMP to decide if these tools are appropriate beyond the vendor's service procedures and to determine appropriate validation techniques for each tool. The QMP may choose to use tools different from the vendor for acceptance testing and/or routine testing. This may be done to standardize equipment across different vendors or to choose equipment that provides more data and/or is easier to use than the vendor supplied device.

In general, the tests in this document do not preclude the use of vendor tests or tools. However, the QMP must use them in such a way that ensures these guidelines are met. Therefore, the QMP must ensure that not only is the appropriate parameter being tested, but that the output is something that can be adapted in a way that satisfies these standards or the facility's adopted standards based on their own risk analysis.

The QMP is directed to the forthcoming TG‐332‐Verification of Vendor Provided Data, Tools, and Test Procedures, in the final stages of development at time of publication, which addresses how to evaluate vendor tools and systems for independence and how to responsibly use these systems routinely.

### 8.5 Automation and scripting

Incorporation of automation and/or scripting into machine QA programs offers several potential advantages including standardization, reduction of variability between users performing the test, and increased efficiency of the entire QA program. The QMP must have a complete understanding of the automated machine QA techniques and any algorithms used in the analysis. With automated technology, the QMP is responsible for assessing risk, commissioning, and routinely monitoring the performance of automated machine QA approaches. MPPG 8 can be adapted for use with automated QA techniques and scripting, but the QMP must ensure that any automated processes are testing the machine parameters in a way that is compatible with the guidance herein. One example is the use of automated EPID or film‐based automated MLC position detection. As the QMP implements such techniques it is imperative to understand the algorithm employed to identify each leaf, the coordinate system for the analysis and how each leaf position is actually determined. Only then can the QMP determine if the automated process is compatible with the MLC position testing described in this document. Once this determination is made, the test should be standardized across the clinic such that naming conventions, templates, setup and test execution are the same, to ensure that the automated process remains compliant with these guidelines everywhere that automation is used.

### 8.6 Use of arrays

Arrays of detectors can be set up quickly and provide efficient measurement of the beam profile. These devices are commonly designed as 2D/3D arrays of diodes or ion chambers available from multiple vendors in several configurations (see Table 2). The spacing between the detectors typically varies between 7 and 10 mm, though newer array dosimeters for stereotactic fields utilize a smaller (e.g., 2.5 mm) detector spacing to improve the spatial resolution. Although most arrays have inherent buildup, detector arrays are often used with solid water to provide the necessary material to reduce electron contamination and better reflect clinical depths. Arrays can provide dose measurements in a phantom provided that appropriate calibration factors are established upon commissioning the device and the appropriate density corrections and/or virtual phantoms are used in the TPS. Additional device calibration is required to create a uniform response among the detectors. Diode‐based 2D arrays possess non‐trivial angular dependence that must be understood and accounted for, if applicable.^43^ Newer devices have been developed that utilize different strategies to produce 3D dose, such as dose scaling using correction factors to recreate volume dose, orthogonal detector systems, or helical detector configurations.^44^, ^45^

**TABLE 2:** List of array‐based dosimeters (adapted with additions^43^, ^46^).

Manufacturer	Device	Type	Notes
Sun Nuclear	MapCHECK 3	Detector array (2D planar)	1527 diodes, 26 × 32 cm, 7.07 mm spacing
Sun Nuclear	SRS MapCHECK	Detector array (2D planar)	1013 diodes, 7.7 × 7.7 cm, 2.47 mm spacing
Sun Nuclear	ArcCHECK	Detector array (2D helical; computational 3D)	1386 diodes arranged in a helical pattern
Sun Nuclear	IC Profiler	Detector array (2D planar)	251 ion chambers, 32 × 32 cm (primary axes), with 45 cm diagonal length.
PTW	OCTAVIUS 1500	Detector array (2D planar/ rotational)	1405 vented ion chambers, 27 × 27 cm, 7.07 mm spacing, ability to rotate w/gantry
PTW	OCTAVIUS 1000 SRS	Detector array (2D planar)	977 liquid filled ion chambers, 10 × 10 cm, 2.5 mm spacing
PTW	STARCHECK	Detector array (2D planar)	527 plane‐parallel vented ion chambers, 26 × 26 cm, 3.0 mm spacing
PTW	STARCHECK maxi	Detector array (2D planar)	707 plane‐parallel vented ion chambers, 40 × 40 cm, 3.0 mm spacing
ScandiDos	Delta4 PHANTOM+	Detector array (2D orthogonal; computational 3D)	1069 diodes total (2 planes), 20 × 20 cm, 5 mm central spacing, 10 mm outer spacing
IBA	myQA SRS	Detector array (2D planar)	105,000 CMOS detectors, 12 × 14 cm, 0.4 mm resolution, no gaps between detectors
IBA	MatriXX	Detector array (2D planar)	1020 vented ion chambers, 24.4 × 24.4 cm, 7.62 mm spacing
Standard Imaging	QA CrossChecker	Detector array (2D planar)	453 vented ion chambers, 5 mm spacing
ScandiDos	Delta4 DISCOVER	Transmission chamber	4040 diodes, 1.5 mm spacing, link with phantom‐based IMRT QA
IBA	Dolphin	Transmission chamber	1513 ion chambers, 40 × 40 cm, 5 mm Spacing
PTW	DAVID	Transmission chamber	Multiwire ion chamber, real‐time, no link to pre‐treatment IMRT QA
Multiple Vendors	On‐board EPID	Onboard amorphous silicon detector w/ software	Software packages using the linac's standard on‐board MV EPID imager

Many of the typical tests used in this document may be adapted for use with the array types described here; indeed, many are challenging to perform *without* an array (e.g., EDW profile measurement, test W3), though it is possible. After commissioning the array, dosimetry tests (profiles, PDD/TPR, etc.) require the QMP to ensure dose measured can be directly compared to the planning system/PRD (beam model and/or calculated beams) when appropriate (annual testing). This requires a comprehensive understanding of how the array works and how doses are calculated in the TPS. Furthermore, it may require modifying the setup to ensure an appropriate field size can be measured (e.g., reducing source‐to‐surface distance (SSD) to accommodate a larger jaw setting). Many routine tests don't require a direct comparison to the PRD but must be linked to one (e.g., a monthly profile measured on an array must reflect absolute deviation from the model profile, either through a device specific baseline directly compared to the TPS or established during annual scans in a water tank compared to the TPS). AAPM's forthcoming TG‐312: Task Group on Acceptance Testing, Commissioning and Periodic Quality Assurance of Ion Chamber and Diode Detector Arrays aims to provide more detail on the required acceptance testing, commissioning, and periodic QA of ion chamber‐ and diode‐based detector arrays.

### 8.7 Maintaining independence of measurement

While Section 6 of this report refers to some of the minimum QMP resources necessary to perform the recommended tests, it is equally important to consider how equipment is being used and to what extent a QA program relies on a single tool or how multiple tools can use the same input. A resourceful physicist may be able to accomplish the majority of testing recommended in this document with a single array device and would be able to meet requirements for all daily, monthly, and annual checks. However, even a minor oversight or miscalibration of the array could introduce systematic errors that would be very difficult to detect using a single device, resulting in severe propagation of error. Using independent dosimetry systems or spot checks of the array would likely identify the error/miscalibration. The QMP must evaluate the QA program for sensitivity to equipment and personnel error and should use redundant systems.

A common QA scheme uses different pieces of equipment for daily, monthly, and annual QA with opportunity for overlap with different personnel (when possible) performing tests to prevent systematic user and/or equipment error. For example, the daily QA schedule for evaluating the beam profile may use a simplified ion chamber array for flatness and symmetry, a separate diode array for monthly evaluation, and a 3D water tank to scan beam profiles fully for annual constancy checks. Action limits and tolerances for each device and test frequency can be set according to the quality of the equipment, time allotted for the test, and user performing the exam. This also allows for a clear path of escalation if an action limit is exceeded; when the daily QA device exceeds the action limit, the more robust monthly array can be used to investigate the findings from the daily array in more detail. If more questions or a discrepancy between measurements remain, a full investigation of the profile can be investigated with the water tank.

The advent of new QA technologies, particularly arrays and analysis of EPID measurements, have allowed for the combination of many tests in fewer measurements with fewer pieces of equipment.^47^ While this approach offers both time and cost savings, careful commissioning of QA equipment and planning for potential equipment malfunction are even more important as the QA program could potentially be susceptible to a single point of failure. Even in a clinic with very limited resources and a single ion chamber, using a different spreadsheet for monthly and annual measurements could help prevent the unchecked corruption of an important clinical tool. If the QMP detects a change in machine performance, it can be difficult to determine whether there is a problem with the machine or the instrument if the measurement cannot be verified by a different piece of equipment. By carefully establishing device baselines/SRDs (Section 7.1.2) and strategically using equipment, the QMP can build in redundant measures that help verify deviations in machine performance and avoid unnecessary intervention.

### 8.8 Performance testing after maintenance

A key component in the overall machine QA program is identifying the requisite testing following servicing or machine parameter adjustment prior to clinical use. Return‐to‐service testing requires a careful balance of covering all potential risks without completely repeating all QA. Moreover, service activities can introduce risk if the repair was not successful or the repair impacted another aspect of machine performance.

Some vendors can provide a list of recommended tests the QMP should perform, depending on the linac system serviced. The QMP must be familiar with any documents that describe responsibilities for vendor field service engineers (FSEs) and customers post‐repair. One example is the replacement of the machine ionization chamber, which may require QMP assessment of beam profiles and output (TG‐51^31^, ^32^). Other situations may only require an output spot‐check. The QMP must create a summary of the return‐to‐service QA for typical machine maintenance activities that includes the vendor recommendations and any other tests the QMP deems necessary, thereby providing consistency in returning a linac to clinical service. This document could also delineate possible minor adjustments that may be necessary or recommended after certain maintenance procedures, such as minor beam steering or output adjustment. Refer to the forthcoming TG‐314, Fault Recovery in Radiation Therapy, currently in draft at the time of this writing, for further guidance on testing after maintenance.

### 8.9 Review of maintenance documents and customer technical bulletins

Review of the maintenance record and customer bulletins from the manufacturer are an important part of ensuring the linac is operating as intended. In addition to the testing addressed in the previous section, the maintenance reports generated by the service engineer must be reviewed promptly to ensure that the QMP is aware of what work has been performed on the machines. For active issues, the QMP must ensure the problem has been addressed satisfactorily and that the proper testing, covered in Section 7.8, has been performed prior to the machine being returned to service. For preventative maintenance, the QMP must review the report to assess the machine's conformance to the manufacturer's specifications and any corrections the service engineer may have made. All reports must be retained to provide a record of machine performance and service record; many states require the retention of such records.

According to Jairam et al., nearly 50% of all radiation therapy device recalls listed in the Food and Drug Administration (FDA) Medical Device Recalls database involved linacs or linac control software.^48^ Therefore, customer technical bulletins or other documents that alert the linac operator to known potential issues or malfunctions of the machine must be reviewed immediately. The QMP must determine if the issue applies to the clinic's specific equipment. If so, the QMP must ensure the manufacturer's recommended action is performed and determine whether any clinic operation must be modified as a result. The issue and corrective action must be disseminated to all affected personnel to ensure that everyone is aware of the potential issue, how it presents, what could be affected, and what actions were or will be taken as a result. Typically, manufacturers require a signed copy of the bulletin to be returned as proof that it was received and read. The QMP must retain a copy as well. It is often convenient to use this copy to obtain signatures after the appropriate staff members have been informed. These records must also be retained.

*Note: Although the details of the commissioning, maintenance, calibration and care of quality assurance equipment is beyond the scope of this document, it is important to note that the concepts addressed in* *Sections* *7.8* *and* *7.9* *apply to the equipment the QMP uses to perform the testing herein. Failure to test QA equipment after maintenance, to review maintenance documentation, technical bulletins, or software release notes after an update could be detrimental to a QA program based on these or other guidelines*.

## 9 LINAC PERFORMANCE TESTS

### 9.1 QMP review of all tests

As part of the design of the performance testing program, the QMP must have sufficient knowledge to understand how test results and beam parameters may be interrelated. For example, if the daily output were to fail, the cause may be due to a change in the beam energy and not a drift in the monitor chamber. The QMP must also understand the linac's ability and limitations in self‐detecting errors. Further, the QMP must evaluate and understand how each piece of equipment and software operates and its proper use, including limitations in measuring capabilities and input/output of data (e.g., while OAFs that can be compared to the TPS are ideal, most morning QA devices only report flatness and symmetry). Once the QMP fully evaluates the operation and use of a piece of equipment, the QMP must commission and calibrate it. This evaluation is crucial both for designing each specific test for which that piece of equipment is used as well as ensuring the device is commissioned and calibrated properly.

Several performance tests are recommended at different frequencies (i.e., daily, monthly, and annually) and are performed by different personnel often using different equipment. Therefore, the QMP must ensure that all tests being performed for a clinical parameter are considered before making any machine adjustments and potentially changing any device baseline or reference values. When a clinical performance parameter exceeds the action limit and needs to be adjusted, it may be necessary to go back several steps in the QA process to ensure that the adjustment does not affect other clinical values. The QMP must be especially mindful of how any adjustments may affect the agreement between the modeled and delivered dose.

The QMP must not only understand each test, each piece of equipment, and each parameter and frequency, but also how each part is connected within the entirety of the QA program. Therefore, the QMP must routinely review all tests and associated equipment, tolerances, and frequencies to ensure the entire QA program is sufficient for the clinical needs.

### 9.2 Recommended tests

Recommended tests are described below in Tables 3, 4, 5, 6, 7, 8. The tests were chosen based on a review of tests presented in TG‐142^3^, focusing on changes in technology and/or techniques in the interval after MPPG 8.a^5^ was published. This built on the previous committee's work but also resulted in the return of some tests excluded in MPPG 8.a. The committee also attempted to address newer techniques and technology not specifically addressed in the original document, as time has allowed more experience to guide testing requirements. The committee also adjusted some action limits based on the experience of the medical physics community after the MPPG 8.a publication. The QMP must implement tests that are relevant to their clinical practice.

**TABLE 3:** Comprehensive review of machine settings.

Item	Test	Frequency	Action limit
C1	Comprehensive Review of Machine Settings	Annually	Same as commissioning/expected

**TABLE 4:** Dosimetry tests.

Item	Test	Frequency	Action limit
D1	Photon and Electron Output Constancy	Daily^a^	3% of device baseline
		Monthly	2% of device baseline
		Annually	1% of nominal TG‐51 output
D2	Photon and Electron Beam Profile Constancy	Daily^a^	2% of device baseline
		Monthly	2% of device baseline
		Annually	2% of TPS OAFs^b^
D3	Electron Beam Energy	Monthly	2 mm equivalent change of R50
		Annual	2 mm measured change of R50
D4	Photon Beam Energy	Monthly	1% of PDD/TPR (relative change in value)
		Annually	1% of PDD/TPR at reference depth
D5	Dynamic Delivery Control	Monthly	2% of open field dose^c^
D6	Photon MU Linearity (Output Constancy)	Annually	2% of nominal
D7	Electron MU Linearity (Output Constancy)	Annually	2% of nominal
D8	Photon Output vs. Dose Rate	Annually	2% of output for TG‐51 dose rate
D9	Photon and Electron Output vs. Gantry Angle	Annually	1% of IEC gantry 0^o^ output
D10	Photon and Electron OAF vs. Gantry Angle	Annually	1% of OAFs at IEC gantry 0^o^
D11	Small Jaw‐Defined Field Size Output Factor	Annually	2% of TPS
D12	Arc Mode (expected MU, degree)	Annually	2% of MU and 2^o^
D13	Special Procedure Mode (TBI/TSET)	Annually	Output: Same as regular beam
			Energy: Same as regular beam
			Profile: Same as regular beam

^a^ Daily checks must be conducted for the energies used that day.

^b^ Or 1% of what was achievable at TPS commissioning, if beam model deviation neared the recommended 2% limit.

^c^ Or 3%/manufacturer's specifications if not used *in lieu* of test D8.

**TABLE 5:** Mechanical tests.

Item	Test	Frequency	Action limit
M1	Localization device	Daily	2 mm
		Monthly	1 mm
M2	Optical Distance Indicator	Daily	2 mm at isocenter
		Monthly	2 mm over clinical range^a^
M3	Jaw Position Indicators	Daily	2 mm per jaw for single field
		Monthly	2 mm per jaw for clinical range of motion or < 2% deviation in output factor
M4	Jaw position vs. gantry angle	Annually	Within specifications^b^
M5	Light to Radiation Field Coincidence	Annually	2 mm per jaw
M6	Gantry/Collimator Angle Indicators	Monthly	0.5^o^
M7	Physical Graticule (Port Film Graticule)	Monthly	2 mm
M8	Cross‐hair Centering	Monthly	1 mm
M9	Treatment Couch Absolute Position readout	Monthly	Abs: 2 mm,1^o^
M10	Treatment Couch Relative Movement Constancy	Monthly	Rel: 1 mm over 10 cm, 0.5^o^ over 3^o^
M11	Radiation Isocentricity (Jaw Radiation Isocenter with Collimator, Gantry and Couch Rotation)	Annually	2 mm diameter^c^
M12	Electron Applicator Collimator Settings/Interlocks	Annually	Same as TPS
M13	Accessory Latches/Interface (All Slots)	Annually	Same as TPS/functional
M14	Robotic couch shift accuracy	Daily	1 mm and 0.5°
		Annual	1 mm and 0.5° under load

^a^ This is a general action limit. The QMP may elect to create case‐specific action limits that may exceed this based on clinical relevance.

^b^ The QMP must be aware of the clinical impact any shift, even within the manufacturer's specifications, may have on treatment.

^c^ For SRS‐SBRT applications, refer to MPPG 9^40^.

**TABLE 6:** Safety tests.

Item	Test	Frequency	Action limit
S1	Door Interlock	Daily	Functional
S2	Door Closing Safety	Annually	Functional
S3	Audio/Visual Monitors	Daily	Functional
S4	Beam‐On Indicator	Daily	Functional
		Annually	All Functional
S5	Anti‐Collision Test	Daily	Functional (one system each day, rotating through each)
S6	Safety Procedures	Determined by QMP	Implemented
S7	Emergency Procedures	Annually	Implemented; emergency features functional

**TABLE 7:** Wedge tests.

Item	Test	Frequency	Action limit
W1	Electronic Wedge Check	Daily	Internal: Functional; Collimator‐Shaped Wedges: 2% of device baseline
		Monthly	2% of device baseline
W2	Physical Wedge Placement Accuracy	Monthly	1 mm
W3	Wedge Profile for 60^o^ Electronic Wedges, All Energies	Annually	2% of TPS OAFs
W4	Wedge Dose for Collimator Shaped Wedges, Selected Angles	Annually	2% of TPS Dose

**TABLE 8:** MLC tests.

Item	Test	Frequency	Action Limit
MLC1	Small MLC‐defined field size output factor	Annually	2% of TPS for 2 × 2 or smaller^a^
MLC2	Leaf Position Accuracy	Weekly	0.5 mm
MLC3	Sliding window/VMAT holistic test	Monthly	2%
MLC4	Radiation Isocentricity (MLC radiation isocenter with collimator, gantry, and couch^b^)	Annually	2 mm diameter^c^

^a^ 5% for 1 × 1 or below.^40^

^b^ Couch need not be tested if tested in M11 or vice versa.

^c^ For SRS‐SBRT applications, refer to MPPG 9.^40^

Tests are grouped together by class of test (e.g., dosimetry, mechanical, etc.) and the clinical parameter tested. The recommended frequency and action limit are listed with each test. Implementation notes on each test follow the tables. These tests were designed with the assumption that the machine being tested is “general purpose,” treating static electrons, 3D, and IMRT/volumetric modulated arc therapy (VMAT). Please see MPPG 9^40^ for SRS/SBRT tests/action limits. The recommended tests are also listed in Appendix II with a column to aid the QMP in determining clinical applicability. The QMP shall decide whether each test applies to their QA program based on the clinical use of the accelerator. To further assist the QMP in developing/maintaining a QA program, Appendix III reorders the tests by testing frequency.

Definitions:

Daily–this frequency implies that a specific test only needs to be done on the day the function is used, unless otherwise noted

Action limits ^*^–all action limits are listed as “within X%” or “within X mm,” and they are listed to mean the action limit must be within ± X% or ± X mm of the PRD or SRD. When an action limit is listed as a percent change from a value (e.g., 2% of PDD), it indicates a relative change from the original value (e.g., 2% of a 66.0% PDD_10_ is not 64.0‐68.0%; it is 64.7–67.3%). These values are intended to indicate limits that, if exceeded, will likely reduce the quality of patient care. Please see Section 9 for how these “hard” action limits differ from “soft” tolerance limits defined by the QMP.

If a test fails, the QMP must evaluate the possible causes for failure and take corrective action, if necessary. Some failures may not be clinically significant for certain types of treatments. If a failure occurs, the QMP must decide if that machine should be removed from service, or if treatments should be limited to certain types and/or number of treatments, etc., based on the severity of the failure, the system that failed, and the types of treatments/disease.

^*^The previous version of MPPG 8 ^5^ *used the term “tolerance,” which has been traditionally used in this context. However, the AAPM has recently shifted to using “action limits,” as seen in TG‐218*,^49^ *instead defining “tolerance” as the “normal operating range of a system performance parameter,” described in* *Section* *9* *in more detail. This is also consistent with similar “action* *levels” terminology of the Canadian Partnership for Quality Radiotherapy (CPQR)*.^50^

#### 9.2.1 Comprehensive review of machine settings

The linac controller contains many definitions that control treatment parameters and machine configuration settings. Important examples include MLC leaf offset positions, collimator settings for electron applicators, etc. For example, an incorrect collimator setting for an electron applicator can cause significant deviations in both output and beam shape. These definitions can have a large dosimetric impact if they are changed and/or do not match TPS settings. Any settings that were established at the time of commissioning of the linac and/or TPS must be reviewed **annually**. In addition, recent ROILs data^17^ revealed that 30% of reported QA incidents were related to updates and upgrades. Further, Jairam et al. reported that analysis of the FDA device recalls database showed that recalls of linac control software is associated with a four‐fold higher rate of instances of incorrect dose, targeting, or volume events than linac defects,^48^ meaning that review and/or testing of any software updates and upgrades for errors is imperative. Finally, the committee recommends a review of any critical parameters that may have changed upon updates/upgrades of the linac and related systems.

#### 9.2.2 Dosimetry tests

##### D1 Photon and electron output constancy

Photon and electron beam output accuracy remains one of the most important parameters in ensuring accurate treatment delivery. Output must continue to be measured daily, monthly, and annually.

**Daily** output checks must be performed on all clinically used beams and must fall within 3% of that dosimetry system's nominal device baseline that correlates to the reference dose output at TG‐51^31^, ^32^ reference conditions. Daily checks may be restricted to the beams in clinical use for that day, at the discretion of the QMP. The QMP should check all beams daily, as daily checks will help identify issues with infrequently used beams and allow the QMP to correct potential issues before that respective beam is used clinically and reduce the chance a non‐checked beam is used that day. If all beams are not checked daily, the QMP must ensure that no beam is used without a daily check. The QMP should also consider decommissioning beams that are not often used. Readings outside this action limit must be reported to the QMP to resolve the discrepancies and determine the appropriate course of action. Most modern linacs can achieve consistent daily outputs well within this 3% limit. Consider implementing tolerance levels as discussed in Section 9.

**Monthly** output checks must be performed on all clinically used beams and must fall within 2% of that dosimetry system's nominal baseline that correlates to the reference dose output at TG‐51 reference conditions. The QMP should establish a lower threshold at which the beam is recalibrated. See Section 9.

**Annual** output measurements must be performed in accordance with TG‐51, a comparable protocol, or successors in water with equipment calibrated by an accredited secondary standards laboratory within the previous two years. Output measured using TG‐51 (or comparable protocol) for each beam must be within 1% of expected dose in the TPS at the reference depth with the same setup conditions. The QMP should set a tolerance within this action limit where the beam is returned to nominal (Section 9). The annual measurement or recalibration must be externally validated, such as by irradiating IROC TLDs/OSLDs, an outside QMP with independent calibrated equipment, or other equivalent methods.^10^

*Note: Once the beams are calibrated using TG‐51, secondary (monthly, if applicable) and tertiary (daily) measurement systems must then be irradiated to establish or confirm baseline output readings that are tied to the primary (absolute) calibration (refer to section* *7.1* *of this report). The QMP may use a secondary measurement system (i.e. solid water based) for monthly output checks or use a water based system as done for annual calibration. A tertiary measurement system (e.g. an array intended for daily output measurements) may be used for daily checks. The QMP must decide on the details of secondary and tertiary measurement systems; their fundamental attribute must be reproducibility*.

##### D2 Photon and electron beam profile constancy

**Daily** profile constancy must be monitored with the clinic's daily measurement device at one or more points along the radial and transverse planes. For clinics that do not possess such a device, a single point away from the central axis must be measured and compared to the central axis (CAX) dose. This point must be at least halfway to the field edge from the central axis (e.g., +2.5 cm X shift for a 10 × 10 cm field). The direction of the shift must be rotated each day to ensure each quadrant of the beam is measured during the course of the week (e.g., +2.5 cm X, −2.5 cm X, +2.5 cm Y, −2.5 cm Y for a 10 × 10 cm field). Daily profile constancy checks must be within 2% of the device/system baseline value established during annual testing.

**Monthly,** the QMP must review the daily off‐axis measurements or measure beam profile shape with another device or method. Profile constancy must be within 2%.

**Annual** measurements of the beam profile must be performed in water or with an array calibrated such that a comparison to the TPS is possible. At minimum, profile measurements must be performed with the largest full field deliverable in the water tank (e.g., 30 × 30 cm for photons, 25 × 25 cm for electrons) at a depth beyond electron contamination for photons (SAD or SSD) and at a clinically appropriate depth for electrons. To use an array, the SDD must be reduced such that a similarly large field is encompassed by the detectors. Agreement of off‐axis points must be within 2% of the central 80% of a beam as compared to the same profile calculated in a virtual water phantom in the TPS. Comparison of the measured and calculated profiles are preferred, but the committee recognizes the current limited availability of tools to compare those datasets easily. The committee recommends that both linac and QA vendors provide tools to enable comparison of measured and TPS calculated profiles. In lieu of a direct profile comparison, a point‐by‐point comparison must be done. At minimum, 4 points (relative to the CAX) must be compared in each field direction: at each 80% full width at half maximum (FWHM) and midpoint from the CAX. Additional field sizes and depths must be measured and compared as well, specifically the smallest field size used clinically (including for dynamic jaw tracking). For field sizes below 3 × 3 cm, see MPPG 9, IAEA Report 483, and TG‐155.^40^, ^51^, ^52^

*Note: The committee recognizes that the threshold for modeling error established by MPPG 5* ^27^ *is 2% for off axis factors. Since the goal is to ensure the linac is producing the same beam that is calculated in the TPS, it is appropriate to retain the same 2% threshold. The committee also recognizes that if, during TPS commissioning according to MPPG 5, off axis factors were near the 2% limit, measurement uncertainty may make it difficult to achieve 2% agreement. In this case, agreement must be within 1% of what was achievable during TPS commissioning*.

*The committee also recognizes the challenges and time requirements of performing this test without an array. To address these challenges, this committee recommends the acquisition of appropriate equipment*.

*Finally, while the committee is emphasizing the direct comparison of beam profiles to the same beam in the TPS, it acknowledges the utility of continuing to track flatness and symmetry in order to facilitate communication with vendors that may still use these metrics for engineering purposes and comparison with historical measurements*.

##### D3 Electron beam energy

**Daily** measurement devices usually measure electron energy constancy relative to a baseline established during TG‐51 calibration. If a clinic has this capability, it should be used.

**Monthly, t**he QMP must review the daily energy constancy measurements, if used. If a daily device is not used to monitor electron energy, the QMP must measure potential changes in energy. All methods should reflect an equivalent change of less than 2 mm for R50.

**Annual** measurements of electron beam energy must be either a point measurement to verify I50/R50 or measurement of a full ionization curve. If the I50/R50 measurement detects a change in energy (> 2 mm), a full depth scan must be performed in water to verify.

##### D4 Photon beam energy

**Daily** measurement devices usually measure photon energy constancy, relative to a baseline established during annual data acquisition. If a clinic has this capability, it must be used.

**Monthly**, the QMP must review the daily energy constancy measurements, if used. If a daily device is not used to monitor photon energy, the QMP must use a method that can evaluate %dd(10)_x_ or equivalent (such as TPR 20/10). This method can be an indirect comparison (e.g., TPR 15/5, other methods), but must be correlated to the PRD at the reference point. The beam quality metric must be within 1% of the SRD established during the annual scans or what is calculated for the same beam in the TPS (PRD).

**Annual** measurements of photon beam energy must be either a two‐point measurement to validate TPR 20/10 or %dd(10)_x_ or a full depth dose curve in water for reference conditions (e.g., 10 × 10 cm field, 100 cm SSD/SAD). Ideally, a full depth dose curve and comparison to values in the TPS should be performed. The beam quality metric must be within 1% of what is calculated at the reference point for the same beam in the TPS (PRD).

*Note: Recent literature indicates that changes in* %dd(10)_x_ *may be too coarse to indicate clinically significant deviation in energy*.^53^ *The committee encourages the QMP to examine the literature to determine if a more rigorous energy test, such as change in profile shape/off axis factors, is necessary. However, given that TG‐51 is the gold standard for absolute dose calibration and beam quality specification*, %dd(10)_x_ *(and TPR 20/10 as a surrogate) is thus also the standard for beam quality/beam energy determination. Therefore, this guideline adopts the %dd(10)_x_ metric established by TG‐51 and refers to TG‐51, WGTG‐51, or its successor(s) should they adopt a more appropriate metric in the future*.

##### D5 Dynamic delivery control (DDC)

VMAT and sliding window IMRT are types of dynamic deliveries routinely used that require the synchronization of the dose rate with other dynamic components of the machine. To produce a dynamic delivery, some combination of MLC position, MLC leaf speed, dose rate, and gantry speed and position are varied throughout the treatment. Patient‐specific QA does not test the full range of these parameters, therefore, a **monthly** test of each of the dynamic control components must be performed for each dynamic beam delivered clinically. Each of these components must be tested either together or individually during dynamic delivery.

Tests have been designed to ensure the machine control of the individual dynamic components or to test them in combination by varying one dynamic control against another. Varian Medical Systems provides a series of tests for dynamic delivery along with the digital imaging and communications in medicine (DICOM) plans needed to execute them and spreadsheets to help with the analysis. In these tests, the gantry speed is varied against the dose rate control in one test and the MLC speed is varied against the dose rate control in another. Elekta provides similar tests at the time of acceptance. The QMP may also design their own fields to test the different elements. For clinics that perform only non‐VMAT dynamic delivery such as sliding window IMRT, a static‐gantry equivalent of the vendor‐supplied DDC tests must be measured. Alternatively, for either VMAT or static sliding window, individual tests for leaf speed, leaf position, and dose rate modulation must be performed. For multiple dynamically delivered energies, all energies must be tested, but may be rotated monthly.

For a combined DDC test, a nonuniform dose delivery indicates a problem with the dynamic control and the QMP must determine which dynamic component has failed. References and manufacturer recommendations indicate that the dynamic fields are able to deliver a dose within 3% of an open beam with the same dose objective. Most modern machines typically can achieve significantly better agreement. A wide range of available detectors, test designs, and analysis software combinations exist that could be used. Software analysis packages available from several vendors significantly increase the efficiency for these tests. The QMP must decide which tests are important for their clinic and may wish to define tighter action limits depending on the sensitivity of each test/machine combination implemented clinically (see Section 9). If a clinic does not use dynamic delivery, dose rate constancy must be evaluated according to D8. If a clinic uses test D5 *in lieu* of D8, the dose agreement must be within 2% of the open field.

*Note: The QMP must be aware that the available vendor‐provided tests may not cover the complete range of clinically used parameters such as dose rate, MLCs used, or energies. It is up to the QMP to decide whether these tests are sufficient for the breadth of treatments available for each machine or to generate more robust test plans. The committee encourages the vendors to provide tests that encompass the entire range of deliverable dose rates, gantry speeds, and other dynamically delivered parameters*.

##### D6 Photon MU linearity (output constancy)

**Annually,** the QMP must test the clinical range of monitor units used for nonsegmented beams and MU/segment for segmented beams. Segmented fields (including step‐and‐shoot IMRT and field‐in‐field) must be tested to determine that the clinically used beam on/off system properly regulates the number of MU delivered per segment. The dose per MU must be within 2% of the dose per MU calculated at calibration for the range of MU settings used clinically.

For static fields, this can be tested by simply varying the MU setting for the reference field. For segmented fields, this can be done by varying the MU/segment down to the minimum clinically allowed and comparing with the reference (static) field of the same size or dose/MU calculated in the TPS.

##### D7 Electron MU linearity (output constancy)

**Annually,** the QMP must test the clinical range of MUs for all clinically used electron beams. The dose per MU must be within 2% of the dose per MU calculated at calibration.

##### D8 Photon output versus dose rate

**Annually,** the clinical range of dose rates, including for static fields or for the range of allowable dynamic dose rates, must be tested to ensure output constancy within 2% of the calibrated dose rate. DDC tests discussed in D5 may be sufficient for testing this parameter.

##### D9 Photon and electron output versus. gantry angle

**Annually,** photon and electron output must be within 1% of output at the gantry head “up” (International Electrotechnical Commision (IEC) 0 degrees) position for all gantry angles. This can be measured with an ion chamber in solid water or an array with orientation changed to match the gantry angle by placing the setup on its “side” on the table or with various chamber/array gantry mounts. Without a gantry mount, setup may be difficult for the head “down” (IEC 180 degrees) position. For this reason, 2% is acceptable at this angle without a gantry mount. Electrons need not be tested at the head down position unless used clinically at angles near IEC 180 degrees.

##### D10 Photon and electron beam profile constancy versus gantry angle

**Annually,** the QMP must test beam profile constancy at multiple angles encompassing the full clinical range of gantry motion: 90, 180, and 270 degrees IEC are convenient to use. A gantry‐mounted array is ideal for this type of measurement, provided rigid positioning of the mount with gantry rotation is verified. Off axis points must match within 1% of values at IEC gantry 0. For electrons, the array may be laid on its “side.” If an array is not available, single point measurements for each beam “quadrant” (+X, ‐X, ‐Y, +Y) must be measured. This should be done at the midpoint off axis for a 20 × 20 cm field (± 5 cm from CAX) since this ensures full scatter is still achieved in a typical solid‐water phantom, as well as being similar to most field sizes measured on arrays. Recognizing the difficulty of performing this measurement without an array at IEC gantry 180 degrees, 2% is allowable for that angle. As in D9, electrons need not be tested at the head down position unless used clinically at angles near IEC 180 degrees.

*Note: The committee also recognizes the challenges and time requirements of performing this test without an array. However, the effect of gravity on the beam‐line components requires such rigorous testing. To address these challenges, this committee recommends the acquisition of appropriate equipment*.

##### D11 Small jaw‐defined field size output factor

**Annually,** the QMP must validate the output factors for the smallest jaw‐defined or jaw/MLC‐defined (for machines with only one set of jaws) field size used clinically, including what is allowed for jaw tracking. For fields below 3 × 3 cm, IAEA TRS‐483, and/or TG‐155 should be consulted for measurement techniques. Output factors must be compared to TPS‐calculated outputs under the same conditions within 2%. Refer to MPPG 9 for small fields used only for SRS/SBRT. Please see test MLC 1 for the equivalent test for MLC‐defined fields.

*Note: This test was previously excluded on the basis that the components that contribute to output factors were already tested individually*.^5^ *While this is technically valid, modern planning techniques (e.g., VMAT and jaw tracking) rely heavily on the accurate definition of all output factors used clinically. At smaller fields especially, the output factor can be dramatically affected by a machine component that is otherwise within the action limit (i.e., jaw calibration within 2 mm). Furthermore, unlike the MLCs, jaws are calibrated via field light and not hard‐coded. Deviation in light or mirror position can cause small jaw calibration errors not as easily detected in other tests. This is further exacerbated by systems that use two separate field lights. Verifying the output factors, especially for the smallest field, serves as a holistic test that is more sensitive than testing any one of these parameters directly*.

##### D12 Arc mode (expected MU, degree)

**Annually,** if arc mode is only used without dynamic delivery and thus test D5 not performed, the QMP must verify that each beam can deliver MUs within 2% of the total MU of the arc and 2 degrees over the entire arc.

##### D13 Special procedures

**Annually,** the QMP must evaluate any procedures that require special delivery techniques produced by the linac. These could be vendor‐included special machine settings, such as total body irradiation (TBI) and total skin electron therapy (TSET) modes found on some machines, as well as any in‐house special techniques. The guidance given here must be adapted by the QMP to evaluate any non‐standard delivery modes used clinically. At a minimum, output, energy and OAFs must be verified for each special procedure mode at the clinical geometry with accessories in place. The action limits are the same as the standard beams, unless not achievable due to the configuration of the special procedure technique. In these cases, the QMP must understand what the machine is capable of under these conditions and clearly communicate the dosimetric uncertainties to the physician. The QMP and physician must determine whether it is acceptable to proceed with using the special procedure mode. Any special procedure mode not maintained for clinical use must be decommissioned.

#### 9.2.3 Mechanical tests

##### M1 Localization device

**Daily**: The accuracy of the device(s) used to position the patients must be evaluated daily. Lasers have traditionally been used for patient positioning but they have increasingly been replaced with daily image‐guided radiation therapy (IGRT) and/or surface‐guided radiation therapy (SGRT). For non‐SRS/SBRT delivery, every positioning system must be accurate to within 2 mm. Image guidance systems are covered in MPPG 2^6^ while SGRT is covered in TG‐302.^54^ If used for final positioning prior to beam on, lasers must align to within 2 mm of the isocenter. If lasers are used for positioning SRS patients, please see MPPG 9^40^ for appropriate guidance.

**Monthly**: The QMP must either monitor the daily evaluation of the positioning devices discussed above, ensuring that they are accurate within the tighter action limit of 1 mm or independently evaluate the accuracy of such systems to 1 mm. Refer to the documents cited above for non‐laser positioning systems.

##### M2 Optical distance indicator

**Daily** checks must include at least a check of the ODI at a single distance if the ODI will be used for clinical and/or QA setup on that day. If the ODI is not used for either, a single point must be checked routinely. For clinical SSD setups the action limit is 2 mm, but the QMP must understand the limitations of the ODI and the clinical needs for the procedure it is used for. Treatment should not be delayed simply because of an out of action limit ODI as long as the QMP is made aware of the failure, understands the clinical significance–if any–it would have on the entire treatment process, and determines it would have no meaningful effect on the treatment.

**Monthly** checks must include multiple readings spanning the entire clinical range over which the ODI is used. Again, the general action limit is 2 mm. More specifically, the action limit should be determined by the QMP based on the clinical relevance of the error, considering type of treatment, localization method, energies used, etc. Similar to the daily check, a failure does not necessarily mean treatment cannot occur as long as the QMP determines it will make no meaningful impact on treatments. However, it must be serviced as soon as possible.

##### M3 Jaw position indicators

**Daily,** the jaw positions must be checked for a minimum of one collimator setting. This can be done for whichever setting is convenient relative to the other daily checks with the light field (i.e., checking the same setting used with a daily QA device that checks dosimetric parameters). Each individual jaw must be checked. Since the daily check is a visual inspection intended to catch gross errors that would meaningfully impact dosimetry for conventional fields, the action limit is 2 mm per jaw.

**Monthly,** jaw positions must be tested against the readout for at least two settings across the clinical range of motion, including the smallest field used clinically (including the smallest allowed field size used in jaw tracking), which is the smallest field that has been calculated in the TPS and verified as accurate according to the methodology outlined in MPPG 5.^27^ This check should be performed using the actual radiation field, such as irradiating film, an EPID, or other device, but can be a visual check with the light field as long as the light field and radiation field have been recently verified as congruent (test M5). Each individual jaw size must be accurate within 2 mm or a cumulative deviation of all jaws that would create a 2% change in field size dependent output factors, whichever is smaller. Jaw size deviations of 2 mm *in each jaw* generally begin creating changes > 2% in field size dependent output factors for field sizes < 3 × 3 cm, even for the highest energies. For clinics that do not use field sizes smaller than 3 × 3 cm or have verified that 2 mm changes in all jaws do not change field size dependent output factors more than 2% for all clinically used field size and energy combinations, a 2 mm action limit is sufficient. For clinics that do use small field sizes such that 2 mm changes in each jaw would cause a 2% or greater deviation in field size dependent output factors, the QMP must also ensure the field size is symmetric within 1 mm. The QMP should consult with a service engineer if the cumulative jaw position error for both jaws in an axis is > 3 mm.

**Annually,** if the actual radiation field is not used for the monthly test (e.g., the light field is used), perform the same test measuring the radiation field defined by the jaws. Each individual jaw size must be accurate within 2 mm or a cumulative deviation of all jaws that would create less than 2% change in field size dependent output factors, whichever is smaller.

*Note: The committee recognizes the wide range of field size definitions, calibrations and jaw action limits used for acceptance testing that exist between vendors and machine type. The QMP must understand the relationship between jaw position and field size and how the jaws are calibrated for each machine type used in the clinic. If the jaw positions do not meet the action limits defined here but are within what is specified by the manufacturer, the QMP must work with the FSE  to determine if the action limit is still achievable and adjustments made accordingly. If it is determined that a particular machine still meets the vendor specifications but, because of mechanical limitations, cannot meet the action limits defined here, the QMP and FSE must determine what is realistic for that machine. Subsequent tests must monitor for any deviation beyond what is deemed reasonable, ensuring that the manufacturer's specifications are always met. In this case, the QMP must understand the limitations of the system and the dosimetric impact such deviations in jaw size would have on each treatment. The types of treatments available on that machine must be limited accordingly*.

*Vendors are also encouraged to recognize the continued link between jaw‐defined field sizes and dosimetric accuracy for clinical techniques that use one or more jaws to define the field edge and refine their jaw action limits accordingly*.

##### M4 Jaw position versus gantry angle

**Annually** or after service, jaw position constancy relative to gravity (colloquially called “slop”) must be assessed for each jaw. Deviation must not be more than the manufacturer's specification. While there is no set action limit here since each machine is limited by machine design, the QMP must understand the clinical impact typical jaw deviations could have on treatment, especially those that use the jaws to define a field edge near an organ at risk. For Elekta users, each jaw and each MLC bank must be tested. Other tests in this document may also show jaw position deviation at various gantry angles and can be used in lieu of this test as long as it measures movement in all jaw/MLC banks in the gravity‐influenced plane.

##### M5 Light to radiation field congruence

The importance of the light field in photon treatments has diminished with the increased use of IGRT, although it is still necessary for the setup of electron beams and some non‐IGRT beams. If the light field is used clinically, used in test M3, or the crosshair and/or light field is used to perform other QA tasks, the light to radiation field congruence must be tested at least **annually** for both **jaws and MLCs**. If the light field is frequently used clinically, the QMP must determine if this test should be performed more frequently. It must also be verified after service to the mirror, field light bulb(s) or any work on the treatment head that may inadvertently affect the bulb or any component of the optical system. The action limit that encompasses all machine types is 2 mm per jaw, but the QMP should determine if a tighter limit is achievable for each machine, especially if the light field is used for other tests.

##### M6 Gantry and collimator angle indicators

**Monthly,** gantry and collimator angle readouts at cardinal angles must be tested. If the imaging system uses a separate gantry encoder, it must be checked as well. Agreement must be within 0.5 degree.

##### M7 Physical graticule

**Monthly,** if the physical graticule is used clinically, the QMP must ensure it is accurate within 2 mm. If the clinic uses only the digital systems (e.g., digital graticule) to localize the treatment center, the QMP must test in accordance with MPPG 2.^6^

##### M8 Crosshair centering

**Monthly,** the mylar crosshair walkout must be tested if it is used to mark the central axis on the patient, the ODI is used for patient setup, or the crosshair is used as a reference to isocenter during QA procedures. The walkout must be within 2 mm, ensuring the crosshair is always within 1 mm of the collimator rotational axis at isocenter.

##### M9 Treatment couch absolute position readout

**Monthly,** the accuracy of the couch's absolute position must be tested for constancy, ensuring no change in the readout of known clinically relevant positions, such as a specific point on the couch verified at isocenter. Accuracy must be within 2 mm and 1 degree.

##### M10 Treatment couch relative movement constancy

**Monthly,** the ability of the table to move a known distance (e.g., 20 cm and 30 degrees) within 1 mm for translational moves and 0.5 degrees for rotational moves for the clinical range must be tested. This can be done by manually adjusting a phantom to a known position away from the reference and then using any clinical positioning system to return the phantom to the correct position, ensuring the relative movement is within 1 mm and 0.5 degrees of the expected shift. If MPPG 2^6^ is followed for the imaging system, the couch shifts are inherently tested and this test is unnecessary, as long as couch rotation is included in the daily testing or tested monthly.

##### M11/MLC 3 Radiation isocentricity (MLC/jaw radiation isocenter with collimator, gantry, and couch rotation)

**Annually,** the radiation isocentricity must be tested to ensure all axes (collimator, gantry, and couch) rotate about the same location within 1 mm. A Winston‐Lutz type test (WL test) that measures all three axes in a single test should be performed (the beam center must not deviate from the isocenter by more than a 1 mm radius for any clinically used collimator/gantry/couch combination), but the committee recognizes that this is not always practical based on machine type and equipment/software available. Therefore, in situations for which a WL test is not feasible, traditional individual star/spoke shots must be performed for each axis. In this case, the runout on individual spoke shot images must circumscribe a circle that is < 2 mm in diameter. The QMP must refer to MPPG 9^40^ or similar document for the frequency and action limit of this test in an SRS/SBRT setting. Jaws and MLCs must be tested. For some machines, this may require repeat testing for each aperture. For Elekta machines where the MLC bank is used as a jaw, there is no need to test both independently. For Varian machines, since jaw and MLC positions are static while rotating the couch, the couch axis only needs to be tested for a single beam shaping device (jaws or MLC). Performing this test with both jaws and MLCs can isolate causes of errors and ensure the congruence of both beam shaping systems, in addition to the isocentricity of the entire delivery apparatus.

If using a WL style test, the QMP must be aware that WL tests can detect errors in multiple components of a linac. For example, a machine with a perfectly aligned radiation center can still fail this test if the jaws are asymmetric. Therefore, it is imperative that the QMP thoroughly analyze the results of the WL test to determine the real cause of any failure.

*Note: The WL test is superior to star/spoke shots and must be performed if the clinic has the capability. If a WL test cannot be performed because the phantom or software is not available, this committee recommends the acquisition of appropriate equipment. If a WL test cannot be performed because of linac limitations (e.g., older Varian “R‐arm” EPID), the QMP must assess what delivery limitations that machine may also have and limit treatments accordingly*.

##### M12 Electron applicator collimator settings/interlocks

**Annually,** each electron cone that is used clinically must be tested for all available energies. The user must attach the cone and verify the machine code for the cone is read correctly and that the jaws drive to the correct positions. Each cone must be checked for physical integrity, as well as touch guards and interlocks including insert detections and coding.

##### M13 Accessory latches/interfaces (all slots)

**Annually,** verify that any accessory that mounts to the linac head latches properly and will not be dislodged or move in a way that will clinically affect the dose distribution as the gantry rotates. This test is included to verify accessories that may not be included in M12 or W2 (e.g., the block tray).

##### M14 Robotic couch shift accuracy

**Daily,** if the robotic couch will be used for high‐risk procedures such as hypofractionation, procedures with close OAR proximity, etc., the ability of the couch to shift all six degrees of freedom accurately within 1 mm and 0.5 degrees must be tested (though SRS/SBRT is not covered here, daily robotic couch testing for these procedures would satisfy this test). This test should be performed at least weekly, even if high‐risk procedures are not performed since robotic angular motion can often be simply incorporated into the imaging system positioning test prescribed in MPPG 2.^6^

**Monthly,** if the robotic couch has not been tested daily or weekly, the ability of the couch to shift all six degrees of freedom accurately within 1 mm and 0.5 degrees must be tested.

**Annually,** the above test must be performed under load. This load must be sufficiently heavy to rigorously test the table, but not necessarily at the vendor maximum load, as to limit undo wear on the table.

*Note: Some vendor provided tests may be adequate to satisfy M14, as long as the QMP fully understands the tests and whether it meets the requirements above. Please refer to the forthcoming TG‐332 for “black box” devices*

#### 9.2.4 S Safety tests

##### S1 Door interlock

**Daily,** the functionality of the door interlock must be checked to ensure that the radiation beam will terminate if the door is opened.

##### S2 Door closing safety

**Annually, t**he QMP must test that the door is able to function in a safe manner when staff and patients enter and exit a treatment room and halts or reverses motion when obstructed. The QMP must test the emergency opening options (e.g., battery backup, come‐along, etc.) for sliding doors or heavy swing doors. The QMP may determine an alternative frequency for this test based on the type of door and its opening design.

##### S3 Audio/visual monitors

**Daily,** the functionality of the audio and visual monitoring systems of the patient must be checked. At least one channel of audio and one channel of video monitoring are required for clinical use of the machine.

##### S4 Beam‐on indicators

**Daily,** the functionality of beam‐on indicators at the console and the door must be checked.

**Annually,** all beam‐on indicators (inside and outside the vault) must be checked.

##### S5 Anti‐collision test

**Daily,** a single anti‐collision device must be checked for system function, rotating through each system on subsequent days such that all devices/interlocks are tested routinely. These include laser guards and touch guards for imaging arms and the EPID. Electron cone touch guards are also checked annually in test M12.

##### S6 Safety procedures

The QMP must use knowledge and experience to determine a set of any additional safety tests needed and the frequency that is necessary to perform them. These tests must be appropriate for the clinical practice and technology used. The QMP may refer to manufacturer's guidelines and/or state regulations to determine which tests are appropriate; however, the QMP must decide how these tests are implemented clinically.

##### S7 Emergency procedures

The QMP must ensure that a written emergency procedure exists and that the back‐up counter, beam off, EPOs, redundant couch drive and other available emergency features are tested annually. Some of these may be tested by service or other personnel, but it is the QMP's job to ensure they are indeed tested. The QMP must also ensure that all emergency systems and/or interlocks required by state law are also tested.

#### 9.2.5 Wedge test

##### W1 Collimator shaped/internal physical wedge check

**Daily,** collimator shaped and internal wedges must be checked for functionality. If daily measurement equipment allows, the output of the steepest wedge angle should be measured to within 2% of the device baseline established annually.

**Monthly,** the QMP must review the daily wedge output results, if performed, to ensure the results were within 2% of expected. If a daily output is not obtained, the output of the steepest wedge angle must be measured to within 2% of the device baseline. If the wedge can be produced by multiple beam shaping devices (e.g., either Y1 or Y2 jaws can be used), each must be tested. Alternatively, the QMP may elect to alternate wedge angles each month in order to rotate through the complete set of available wedges, as long as the QMP understands the decreased sensitivity of the test at lower angles. Rotating the wedges monthly may satisfy test W4, as long as the measurement can be compared directly to the PRD.

##### W2 External physical wedge placement accuracy

**Monthly,** verify external physical wedge placement on the accessory tray, tray placement, and latching position on the treatment head. A scribe mark on the wedge, tray, and tray slot can be used to verify repeatable positioning of the wedge. Test all wedges that are used clinically. Wedge placement must be consistent within 1 mm at the accessory tray.

##### W3 Wedge profile for steepest collimator shaped/internal physical wedges, all energies

**Annually,** a minimum of the steepest wedge angle used clinically must be measured for all energies and off‐axis points within the central 80% of the beam compared to the same beam calculated in the TPS. For techniques that can use more than one movable jaw (e.g., Varian's EDW Y1 or Y2 for in or out), each moveable jaw must be tested. Agreement must be within 2% for all points.

##### W4 Wedge dose for collimator shaped/internal physical wedges, selected angles

**Annually,** the dose in wedged fields must be measured for a subset of clinically commissioned collimator shaped and internal physical wedge angles for all energies (including in/out if a different jaw is used). This may be done using absolute dose or wedge factors, but must be compared to the same beam in the TPS to within 2%.

#### 9.2.6 MLC Multileaf collimator tests

##### MLC 1 Small MLC‐defined field size output factor

**Annually,** the QMP must validate the output factor for a small MLC‐defined field size, 2 × 2 or smaller. The output factor for the measured field must be within 2% of the same beam measured in the TPS (see MPPG 9^40^ for fields smaller than 2 × 2). The QMP is encouraged to test the smallest field possible with the available equipment, using guidance from documents such as MPPG 9^40^, TG‐155^52^, TRS‐483^51^, etc. When performing such measurements, the QMP must understand the limitations of available equipment when lateral charged particle equilibrium begins to break down, applying appropriate correction factors or recognizing that the available device is unsuited for such measurements. In the latter case, the QMP must decide how the inability to measure these small fields accurately will affect clinical cases, while advocating for the appropriate equipment.

##### MLC 2 Leaf position accuracy

**Weekly,** ^55^ quantitative positional accuracy of all leaves (and backup jaws, if applicable) must be checked to ensure leaves move to prescribed positions to within 0.5 mm^55^ for clinically relevant positions^*^. The test must be performed at different gantry angles or in arc mode to detect any gravity‐induced positional errors. An acceptable test includes a quantitative picket‐fence type test, though more rigorous testing may be necessary, based on clinical requirements. **Previously recommended visual tests are insufficient to detect non‐negligible errors**, however visual testing may be sufficient if an appropriate quantitative reference is available such as including a reference MLC offset with a known deviation representing the 0.5 mm action limit.^55^ Other tests that are tailored to the design of Elekta and Siemens MLC systems also exist (Hancock and Diamond jig, respectively). It is the responsibility of the QMP to understand the MLC positioning system and decide which test is appropriate based on clinical need. At this time, log file analysis alone is not an adequate replacement for testing that measures the radiation field to detect leaf position.^56^ The QMP must also ensure that the MLC system performs consistently from week to week by monitoring the trend of the MLC positions measured during this test.

^*^If a vendor and the QMP together determine that a particular machine cannot meet this action limit, an action limit must be determined, in writing, which is within the manufacturer's specification as close as reasonably achievable to 0.5 mm. The facility must determine, in writing, what limitations, if any, this places on the types of treatments the machine is capable of performing.

##### MLC 3 Sliding window/VMAT holistic test

**Monthly,** Clinics that use sliding‐window and/or VMAT techniques must perform a test similar to the ion chamber portion of the VMAT/IMRT test 7.4 from MPPG 5, Table 8,^27^ on a monthly basis (this may satisfy a portion of the test described in section 8.3). This test delivers a clinically relevant complex VMAT or IMRT plan to a phantom with appropriately small dosimeter^*^ placed in the target area. The dose in the target region must be within 2%^**^ of what is calculated in the treatment planning system. As this test is intended to assess continuously moving MLCs and not beam generation, only one energy need be measured. Machines performing VMAT and sliding‐window IMRT need only test VMAT to satisfy this test.

^*^An appropriately small ionization chamber, scintillator or similarly small detector must be used to limit the variables in the setup. It is important to directly compare dose measured with the dosimeter to the dose calculated at the same point in the TPS as analyses that use DTA (or gamma) can be too forgiving for an adequate assessment.

^**^ See note in section 8.3

##### MLC 4 Radiation isocentricity (MLC radiation isocenter with collimator, gantry, and couch rotation^*^)

See M11

^*^Couch may be tested for MLC or Jaws

*Note on MLC QA: The QMP must be aware of the current evolving relationship between MLC QA and patient‐specific quality assurance (PSQA). Data suggest that traditional PSQA is insufficient at detecting errors and that the “frequency and vigor of linac MLC QA tests” must increase*.^57^ *It is very likely that most clinics require more rigorous MLC QA than presented here. However, the committee has presented guidance based on literature‐supported increased frequency and rigor of previously recommended tests*,^3^, ^5^, ^55^, ^56^ *equipment/software commercially available to perform the tests, and the current lack of consensus on what said “rigor and frequency” looks like. The QMP and vendors alike are encouraged to monitor the literature regarding this potential paradigm shift. Vendors should provide improved MLC position testing capabilities in sliding window‐type techniques, as this is a known deficiency in MLC testing*.

### 9.3 End‐to‐end testing

As technology has advanced in the field of radiation oncology, so too has the integration of steps in the treatment process. What were somewhat isolated silos connected by clearly defined bridges, simulation, treatment planning, localization, and treatment have increasingly become interconnected, with each phase no longer completely distinct. A clear example of this integration trend can be seen in the emerging area of adaptive planning. Therefore, it is no longer sufficient only to test each subsystem or process separately.

While MPPG 8 specifically addresses linac quality assurance, other documents address quality measures of simulation, image registration, contouring, treatment planning, secondary dose calculations, IGRT, and other aspects of treating a patient with radiation. Each of these documents aim to provide guidance as to what the acceptable error and uncertainty are for each particular portion of the treatment process. However, following these documents do not characterize the uncertainties for the entire workflow of simulation to treatment; from end to end (E2E).

The only way to quantify that the entire treatment process provides an acceptable end result is to test the workflow from end to end. E2E testing uses a phantom to simulate a patient going through the entire treatment process–simulation, registration, contouring, treatment planning, secondary dose calculation, PSQA, target localization (often with IGRT), and delivery, with a quantitative dose result from the analysis of the resulting dose delivery. Plans created must be similar to clinically delivered plans that push the system in its allowed operating limits. Given that this process is intended to simulate a patient, each team member should perform their typical function in the process.^40^ This ensures that any process deficiencies can be identified and distributes the workload similar to a clinical case. Further, any ancillary positioning systems must also be tested during the E2E process (e.g., SGRT). To adequately develop this process, QMPs must be provided the appropriate equipment to perform an E2E test that can assess these systems in an accurate and efficient manner.

Ideally, the delivered dose should be analyzed by a third‐party independent phantom service (and must be as part of the commissioning process). Several entities provide this service and often provide a set of planning goals that challenge the system relative to the treatment location. If there are no significant changes in process, mechanics, algorithms, machine parameters, etc., from commissioning, a third‐party analysis may not be necessary if cost or logistics is prohibitive. In the absence of an independent phantom, an in‐house system must be used by the QMP to evaluate the dosimetry. 2D systems (planar film, arrays) used in a phantom of solid water or pseudo‐3D methods (like orthogonal films in a phantom) can be acceptable, if the limitations are known and the system is static (i.e., it remains in the same position as an actual patient, not rotating with the gantry). **At a minimum, the QMP must oversee an annual E2E test** ^10^ that ensures the dose delivered from a complex, clinically used technique with an appropriate dosimeter inserted into a phantom at a single point is within 5% of what is calculated in the TPS *. This test should be similar to the E2E test from MPPG 5, Table 8.^27^ Any deviation of 3% or more must be investigated to determine if improvements can be made that would allow better agreement. More rigorous testing as described above should be pursued, if feasible. The QMP may consider additional E2E tests for each different clinical process, such as gating, surface guidance, dynamic conformal arcs, etc. If additional E2E tests are performed and a third‐party service is used it is likely feasible to only test one treatment process per machine while using an in‐house system for other processes. In this case, the most complex treatment technique used clinically should be tested using the third‐party service. Given the multiple phantoms, dosimetry systems, and techniques with which more rigorous E2E testing can be performed, it is impossible to provide a specific action limit for such tests, but the general 5% limit above must be adapted accordingly. It is the QMP's responsibility to understand the results of an E2E test and how it relates to clinical delivery. Third party services typically provide their own limits and other limits are available in the literature.

The committee recognizes that many systems and processes that comprise a full E2E test are not technically a part of machine quality assurance. However, these other systems depend on the linac performing appropriately. All of these systems and processes work in concert, contributing to the cumulative uncertainty of the treatment. As the complexity of radiotherapy delivery has increased, so has the blending of the traditional silos of simulation, treatment planning, patient alignment and machine performance. E2E tests can serve as a bridge between these different portions of the treatment delivery process, and, therefore, serve an important role in machine performance testing and the overall quality assurance program.

Finally, it is imperative that the QMP and other clinical stakeholders recognize that any E2E test, whether via a third party or in‐house, is not a comprehensive test of the beam delivery system. It is a holistic test of processes in which beam delivery is only the last step. The measurements made during this test give only a broad sense of how the linac performs, especially since errors induced by other parts of the treatment process can exacerbate–or even mask–deviations in final dose agreement. Ultimately, an E2E test cannot replace any testing of specific parameters described in section 8 (with the exception of the recommended testing for sliding‐window techniques in MLC3). In fact, comprehensive linac performance testing will reduce the influence that beam delivery has on any deviation in the end result of an E2E test, allowing the QMP to focus on improvement in other areas of the process.

^*^If the E2E test is intended to include the ion chamber test for sliding window‐type delivery in test MLC3, the limit is 2% (or within 1% of what was achievable at commissioning) to ensure that the dynamic MLC component of the test is also within specifications according to MLC2/MPPG 5.^27^

## 10 QMP GUIDANCE ON TOLERANCE AND ACTION LEVELS

The ability to identify when a parameter is outside the typical operating range, but not impacting patient care allows timely intervention without the need to take the system out of clinical service. Tolerance limits define bounds of the normal operating range, which is an allowable expected variation in a performance metric. Tolerance limits represent the intrinsic performance of a system and can be set based on vendor guidance or defined by statistical process control chart limits produced from trending test‐to‐test variations. Action limits, however, are clinically relevant thresholds that if exceeded may significantly impact patient care. If a tolerance limit is exceeded but still within the action limits defined in this document, it is safe to continue clinical use of the equipment, but the source of deviation requires timely investigation and intervention. This creates a “warning range” between action and tolerance levels in which the equipment can be returned to the normal operating range without need to take the equipment out of clinical operation.^49^, ^50^ The QMP must determine the appropriate time limit that a machine can be used with a parameter in this “warning range,” based on clinical use and potential treatment effects to ensure quality care is still received. The QMP should collaborate with the physician, therapists and other members of the treatment team in this determination, as well as discuss possible mitigation strategies until the machine can be returned to nominal. Risk analysis, such as presented in TG‐100^12^ can assist in these determinations. The QMP must also look at the pattern of results relative to the tolerance limits to assess if systematic trends exist and if the trend is projected to reach the limit and take appropriate corrective actions. Ultimately, the committee encourages the use of tolerance levels, as most modern equipment is consistently able to achieve better results than the limits presented here. Tolerance levels help ensure the care received is the best possible with the equipment available, not just at the minimum guidelines.

## 11 SUMMARY

The committee sought to build on MPPG 8.a^5^ by addressing technological and technique changes that occurred since initial publication by clarifying the idea of baseline and primary reference dataset, improving certain tests and limits based on more recent information, broadly addressing the adoption of more widely accepted QA tools, and adjusting the frequency and target values of some tested parameters. This guideline encompass a wide range of clinical configurations and equipment.

The QMP is responsible for overseeing the development of a comprehensive QA program for their equipment, considering the specific modalities, types of treatments, diversity of patients and the type and frequency of patient alignment used in their clinic. Ultimately, this guideline provides guidelines the QMP must use in selecting the tests and action limits used for their linear accelerators. A more rigorous program will likely be developed if risk analysis techniques are employed as suggested in Section 5.3 and TG‐100^12^ but even after performing robust FMEA, the guidelines presented here serve as a floor for the acceptable level of QA that would ensure safe and high‐quality radiation treatments.

## AUTHOR CONTRIBUTIONS

This guideline was reviewed and updated by Medical Physics Practice Guideline Task Group 364 of the Professional Council of the AAPM. Each author reviewed recent literature on the topic and offered opinions on and language for the guideline. They also reviewed and applied comments from the full AAPM membership to the document.

## CONFLICT OF INTEREST STATEMENT

The Chair of Medical Physics Practice Guideline 8.b: Linear accelerator performance tests (TG364) has reviewed the required Conflict of Interest statement on file for each member of TG364 and determined that disclosure of potential Conflicts of Interest is an adequate management plan. Disclosures of potential Conflicts of Interest for each member of TG364 are found at the close of this document.

Members of TG364 listed below attest that they have no potential Conflicts of Interest related to the subject matter or materials presented in this document: Salim Balik, Eileen Cirino, Austin Hadley, Navneeth Hariharan, Chaitanya Kalavagunta, Robert F Krauss, Hossein Lavvafi, Kiernan T McCullough, John P Sawyer, Koren Smith, Jeff D Winter, Binbin Wu, Nicholai Wingreen

Members of TG364 listed below disclose the following potential Conflict(s) of Interest related to subject matter or materials presented in this document: Shannon M Holmes is employed by Standard Imaging, Kayla N. Kielar is employed by Varian Medical Systems, Steven Palefsky is employed by Elekta, Daniel M. Ritt is employed by Radiological Imaging Technology, Jason B. Tracy is employed by Sun Nuclear.

## APPENDIX I TG‐100 AND LINAC QA: ADDITIONAL CONSIDERATIONS

From the Workgroup on the Implementation of TG‐100

**Summary**:

This document provides a context for additional considerations in designing a quality management system. The document also provides references to existing tools and resources.

How to score quick wins?

*Reference*: https://www.aapm.org/QualitySafety/TG100/documents/PracticalSuggestionsTG100.pdf

1. Start with a small project
2. Assemble a team representative of the entire department relevant to the project, and educate the group on prospective risk analysis concepts and the TG‐100 recommendations. Educational videos are available at https://www.aapm.org/QualitySafety/TG100/ImplementationGuide.asp
3. Focus on process mapping first (see www.draw.io for some mapping tools)
4. Focus the initial Failure Modes and Effects Analysis (FMEA) work on a clinical process—not on established machine QM processes (sample process steps and FMEA tools are available in the TG‐100 Repository at mpec.aapm.org)
5. Invest time and effort to become proficient at the FMEA method and to develop useful tools for further FMEAs
6. Build a Fault Tree Analysis based on your process maps
7. Design your quality management program using the knowledge gained, as described in the TG‐100 report
8. Any significant changes to the quality management program should be undertaken only after careful review.

Available tools and resources—https://mpec.aapm.org/repository/home.php

**Reasons for risk analysis**:

After more than a decade of work and internal review and deliberation, the AAPM's Task Group 100 report was published in the journal *Medical Physics* in July 2016.^12^ The report constitutes the AAPM's endorsement of a paradigm shift in the approach to quality management in radiation oncology.

The TG‐100 report recommends the adoption of four risk analysis tools toward risk‐informed development of radiotherapy quality management programs: process mapping, failure modes and effects analysis, fault tree analysis and an organized, stepwise process for addressing identified weaknesses and designing quality‐management procedures. Together, these tools, when properly adapted to radiation oncology, provide a framework for identifying relative risks in each clinic's radiation oncology program. This information would allow for risk‐mitigation interventions that would then be monitored for effectiveness, forming a continuous feedback loop. This Appendix to MPPG 8.b aims to provide guidance regarding the incorporation of the TG‐100 recommendations in designing a clinic's linac QA program.

Simply put, the core premise behind the TG‐100 recommendations is that risk must be assessed in the context of the practice setting, and that each clinical program has a unique combination of practice considerations, including, but not limited to, technology; staff schedules and experience; scope of clinical procedures; institutional conventions; and regulatory considerations. TG Reports and MPPGs are *national* recommendations and guidelines intended for adoption by Qualified Medical Physicists (QMPs) as they deem appropriate for the local practice setting.

*So, how could one use the TG‐100 recommended tools to design a linac QA program that is appropriate for the local practice environment*? We'll use two hypothetical practice settings to illustrate how the TG‐100 recommended tools can help in this regard:

- ***Clinic A***: Single‐linac freestanding clinic owned by a urology practice, with the radiotherapy service exclusively focused on moderately fractionated prostate external beam therapy. The center houses a linear accelerator with high‐resolution MLC, 6DOF couch, on‐board x‐ray imaging and optical surface imaging.
- ***Clinic B***: Single‐linac department in a community hospital, with a broad radiotherapy service consisting of conventional 3DCRT/IMRT treatments as well as an active SRS‐SBRT service including single‐isocenter multi‐target cranial SRS. The center's linac configuration is identical to Clinic A's.

Possible approach to enriching the QM in your clinic:

The QMP in each clinic starts by **mapping** the treatment delivery process, working closely with the clinical team. Clinic A has only one process and this results in a single treatment delivery process map consisting, for example, of 25 process steps. Clinic B uses seven separate processes (electrons, 3DCRT without motion management, 3DCRT with DIBH, IMRT/VMAT [4DITV or no motion], SBRT without motion management, SBRT with breath hold, and cranial SRS), with the number of process steps varying from 21 to 53. The QMP selects one of the processes to address. **The TG‐100 recommendation is to keep the initial project small** and not attempt to tackle all the treatment processes at once.

For Clinic A, the target volume is a consistently moderate size and close to the treatment isocenter, and with 20 or more treatment fractions for all patients. For Clinic B, the target volumes range from very large (e.g., whole pelvis) to very small (e.g., cranial SRS), with many treatments subject to significant respiratory target motion and some treatments (e.g., single‐isocenter multi‐target SRS) highly sensitive to rotational misalignment. Active motion management during treatment delivery depends on an interface between the linac control system and a separate system.

Next, the QMP in each clinic works with the clinical team to **postulate potential failure modes for each process step**, as well as potential causes of failure coupled with the “worst‐case clinically feasible” severity, using Failure Modes and Effects Analysis (FMEA). This analysis can either include accounting for existing process controls if only enhancements of the existing QM program is desired, or can ignore existing controls if optimization of QM efficacy and efficiency are the goal. The FMEA results in calculated Risk Priority Numbers (RPNs) for each process step.

Next, the QMP in each clinic works with the clinical team to trace the pathway of the identified potential failures using the Fault‐Tree Analysis (FTA) methodology. For Clinic A, the relatively simple and highly consistent process results in fewer potential failure pathways, whereas for Clinic B, the more complex clinical approaches and combinations of technology (motion management, robotics, x‐ray, and optical surface imaging) result in many more potential failure pathways. [The TG‐100 Repository contains a process mapping and FMEA template tool, and is intended to facilitate sharing of TG‐100 tools within the medical physics community. A concise summary of recent peer‐reviewed articles using the TG‐100 tools to evaluate SRS, brachytherapy and other processes is one of the Repository submissions. The Repository can be accessed at https://mpec.aapm.org/repository/home.php.]

Although both clinics have the same linac configuration, the overall scope of the equipment QA program in Clinic A could be more limited (e.g., the center does not use motion management and the optical surface imaging system is only used as a patient identification tool), whereas the equipment program in Clinic B would need to be comprehensive in scope (all technological components play a crucial role in treatment delivery) and the tolerances for many components may need to be more stringent than the same components in Clinic A (e.g., 6DOF rotational accuracy).

Having worked closely with the center's clinical team to understand how the linac configuration and treatment delivery technology are used (process mapping), what could go wrong (FMEA) and how it could go wrong (FTA), each clinic's QMP would now be in a much better position to design an appropriate linac QA program for their center and to clearly articulate the rationale for the QA program. Incident learning and annual program reviews would provide additional perspective over time, supporting future program refinements.

## APPENDIX II TABLE FOR APPLICABILITY ASSESSMENT

Table AII

**TABLE AII:** Tests with applicability checklist.

Item	Test	Frequency	Action Limit	Applicable to Clinical Practice? (Y/N)	QMP Initials
**Comprehensive Review of Machine Settings**					
C1	Comprehensive Review of Machine Settings	Annually	Same as commissioning/expected		
**Dosimetry Tests**					
D1	Photon and Electron Output Constancy	Daily*	3% of device baseline		
		Monthly	2% of device baseline		
		Annually	1% of nominal TG‐51 output		
D2	Photon and Electron Beam Profile Constancy	Daily^a^	2% of device baseline		
		Monthly	2% of device baseline		
		Annually	2%^b^ of TPS OAFs		
D3	Electron Beam Energy	Monthly	2 mm equivalent change of R50		
		Annually	2 mm measured change of R50		
D4	Photon Beam Energy	Monthly Annually	1% of PDD/TPR (relative change in value) 1% of PDD/TPR at reference depth		
D5	Dynamic Delivery Control	Monthly	2% of open field dose^c^		
D6	Photon MU Linearity (Output Constancy)	Annually	2% of nominal		
D7	Electron MU Linearity (Output Constancy)	Annually	2% of nominal		
D8	Photon Output vs. Dose Rate	Annually	2% of output for TG‐51 dose rate		
D9	Photon and Electron Output vs. Gantry Angle	Annually	1% of IEC gantry 0^o^ output		
D10	Photon and Electron OAF vs. Gantry Angle	Annually	1% of OAFs at IEC gantry 0^o^		
D11	Small Jaw‐Defined Field Size Output Factor	Annually	2% of TPS		
D12	Arc Mode (expected MU, degree)	Annually	2% of MU and 2^o^		
D13	Special Procedure Mode (TBI/TSET)	Annually	Output: Same as regular beam Energy: Same as regular beam Profile: Same as regular beam		
**Mechanical Tests**					
M1	Localization device	Daily	2 mm		
		Monthly	1 mm		
M2	Optical Distance Indicator	Daily	2 mm at isocenter		
		Monthly	2 mm over clinical range^d^		
M3	Jaw Position Indicators	Daily	2 mm per jaw for single field		
		Monthly	2 mm per jaw for clinical range of motion or < 2% deviation in OAFs		
M4	Jaw position vs. gantry angle	Annually	Within specifications^e^		
M5	Light to Radiation Field Coincidence	Annually	2 mm per jaw		
M6	Gantry/Collimator Angle Indicators	Monthly	0.5^0^		
M7	Physical Graticule (Port Film Graticule)	Monthly	2 mm		
M8	Cross‐hair Centering	Monthly	1 mm		
M9	Treatment Couch Absolute Position readout	Monthly	Abs: 2 mm,1^o^		
M10	Treatment Couch Relative Movement Constancy	Monthly	Rel: 1 mm over 10 cm, 0.5^o^ over 3^o^		
M11	Radiation Isocentricity (Jaw Radiation Isocenter with Collimator, Gantry, and Couch Rotation)	Annually	2 mm diameter		
M12	Electron Applicator Collimator Settings/Interlocks	Annually	Same as TPS		
M13	Accessory Latches/Interface (All Slots)	Annually	Same as TPS/functional		
M14	Robotic couch shift accuracy	Daily	1 mm and 0.5°		
		Annually	1 mm and 0.5° under load		
**Safety Tests**					
S1	Door Interlock	Daily	Functional		
S2	Door Closing Safety	Annually	Functional		
S3	Audio/Visual Monitors	Daily	Functional		
S4	Beam‐On Indicator	Daily	Functional		
		Annually	All Functional		
S5	Anti‐Collision Test	Daily	Functional (single point for system function, rotated)		
S6	Safety Procedures	Determined by QMP	Implemented		
S7	Emergency Procedures	Annually	Implemented; emergency features functional		
**Wedge Tests**					
W1	Electronic Wedge Check	Daily	Internal: Functional; Collimator Shaped Wedges: 2% of device baseline		
		Monthly	2% of device baseline		
W2	Physical Wedge Placement Accuracy	Monthly	1 mm		
W3	Wedge Profile for 60‐degree Electronic Wedges, All Energies	Annually	2% of TPS OAFs		
W4	Wedge Dose for Collimator‐Shaped Wedges, Selected Angles	Annually	2% of TPS Dose		
**MLC Tests**					
MLC1	Small MLC‐defined field size output factor	Annually	2% of TPS for 2 × 2 or smaller		
MLC2	Leaf Position Accuracy	Weekly	0.5 mm		
MLC3	Sliding window/VMAT holistic test	Monthly	2% of TPS		
MLC4	Radiation Isocentricity (MLC radiation isocenter with collimator, gantry, and couch^f^)	Annually	2 mm diameter^g^		

^a^ Daily checks should be conducted for the energies used that day.

^b^ Or 1% of what was achievable at TPS commissioning, if beam model deviation neared the recommended 2% limit.

^c^ Or 3%/manufacturer's specifications if not used to test D8.

^d^ This is a general action limit. The QMP may elect to create case‐specific action limits that may exceed this based on clinical relevance.

^e^ The QMP must be aware of the clinical impact any shift, even within the manufacturer's specifications, may have on treatment.

^f^ Couch need not be tested if tested in M11 or vice versa.

^g^ For SRS‐SBRT applications, refer to MPPG 9.

## APPENDIX III TABLE OF TESTS ORDERED BY FREQUENCY

### III.1

Table AIII

**TABLE AIII:** Tests by frequency.

Item	Test	Action Limit	Comment
**Daily Tests**			
D1	Photon and Electron Output Constancy	3% of device baseline	For energies used that day
D2	Photon and Electron Beam Profile Constancy	2% of device baseline	For energies used that day
M1	Localization device	2 mm	
M2	Optical Distance Indicator	2 mm at isocenter	
M3	Jaw Position Indicators	2 mm per jaw for single field	
M14	Robotic Couch Shift Accuracy	1 mm and 0.5^o^	
S1	Door Interlock	Functional	
S3	Audio/Visual Monitors	Functional	
S5	Anti‐Collision Test	Functional (single point for system function, rotated)	
W1	Electronic Wedge Check	Internal: Functional; Collimator Shaped Wedges: 2% of device baseline	
**Weekly Tests**			
MLC2	Leaf Position Accuracy	0.5 mm	For clinics using sliding window/VMAT techniques, performing the VMAT ion chamber test from MPPG 5, Table 8^25^ on a monthly basis is strongly encouraged; dose agreement should be 2%
**Monthly tests**			
D1	Photon and Electron Output Constancy	2% of device baseline	All energies
D2	Photon and Electron Beam Profile Constancy	2% of device baseline	All energies
D3	Electron Beam Energy	2 mm equivalent change of R50	All energies
D4	Photon Beam Energy	1% of PDD/TPR (relative change in value)	All energies
D5	Dynamic Delivery Control	2% of open field dose	Or 3%/manufacturer's specifications if not used to test D8
M1	Localization device	1 mm	
M2	Optical Distance Indicator	2 mm over clinical range	This is a general action limit. The QMP may elect to create case‐specific action limits that may exceed this based on clinical relevance
M3	Jaw Position Indicators	2 mm per jaw for clinical range of motion or < 2% deviation in OAFs	
M6	Gantry/Collimator Angle Indicators	0.5^o^	
M7	Physical Graticule (Port Film Gradicule)	2 mm	
M8	Cross‐hair Centering	1 mm	
M9	Treatment Couch Absolute Position readout	Abs: 2 mm, 1^o^	
M10	Treatment Couch Relative Movement Constancy	Rel: 1 mm over 10 cm, 0.5^o^ over 3^o^	
W1	Electronic Wedge Check	2% of device baseline	
W2	Physical Wedge Placement Accuracy	1 mm	
MLC3	Sliding window/VMAT holistic test	2% of TPS	Similar to test 7.4 from Table 8 in MPPG 5.b^27^
**Annual tests**			
C1	Comprehensive Review of Machine Settings	Same as commissioning/expected	
D1	Photon and Electron Output Constancy	1% of nominal TG51 output	
D2	Photon and Electron Beam Profile Constancy	2% of TPS OAFs	Or 1% of what was achievable at TPS commissioning, if beam model deviation neared the recommended 2% limit
D3	Electron Beam Energy	2 mm measured change of R50	
D4	Photon Beam Energy	1% of PDD/TPR at reference depth	
D6	Photon MU Linearity (Output Constancy)	2% of nominal	
D7	Electron MU Linearity (Output Constancy)	2% of nominal	
D8	Photon Output vs. Dose Rate	2% of output for TG‐51 dose rate	
D9	Photon and Electron Output vs. Gantry Angle	1% of IEC gantry 0^o^ output	
D10	Photon and Electron OAF vs. Gantry Angle	1% of OAFs at IEC gantry 0^o^	
D11	Small Jaw‐Defined Field Size Output Factor	2% of TPS	
D12	Arc Mode (expected MU, degree)	2% of MU and 2^o^	
D13	Special Procedure Mode (TBI/TSET)	Output: Same as regular beam Energy: Same as regular beam Profile: Same as regular beam	
M4	Jaw position vs. gantry angle	Within specifications	The QMP must be aware of the clinical impact any shift, even within the manufacturer's specifications, may have on treatment
M5	Light to Radiation Field Coincidence	2 mm per jaw	
M11	Radiation Isocentricity (Jaw Radiation Isocenter with Collimator, Gantry and Couch Rotation)	2 mm diameter	Couch need only be tested with M11 or MLC3; For SRS‐SBRT applications, refer to MPPG 9^40^
M12	Electron Applicator Collimator Settings/Interlocks	Same as TPS	
M13	Accessory Latches/Interface (All Slots)	Same as TPS/functional	
M14	Robotic couch shift accuracy	1 mm and 0.5° under load	
S2	Door Closing Safety	Functional	
S4	Beam‐On Indicator	All functional	
S7	Emergency Procedures	Implemented; emergency features functional	
W3	Wedge Profile for 60‐degree Electronic Wedges, All Energies	2% of TPS OAFs	
W4	Wedge Dose for Collimator‐Shaped Wedges, Selected Angles	2% of TPS Dose	
MLC1	Small MLC‐defined field size output factor	2% of TPS for 2 × 2 or smaller	5% for 1 × 1 or below
MLC3	Radiation Isocentricity (MLC radiation isocenter with collimator, gantry, and couch)	2 mm diameter	Couch need only be tested with M11 or MLC3; For SRS‐SBRT applications, refer to MPPG 9^40^
**Other Tests**			
S6	Safety Procedures	Implemented	Review safety procedures as determined by QMP
